# Transcriptome analysis of the biofilm formed by methicillin-susceptible *Staphylococcus aureus*

**DOI:** 10.1038/srep11997

**Published:** 2015-07-07

**Authors:** Xiaojuan Tan, Nan Qin, Chunyan Wu, Jiyang Sheng, Rui Yang, Beiwen Zheng, Zhanshan Ma, Lin Liu, Xinhua Peng, Aiqun Jia

**Affiliations:** 1 School of Environmental and Biological Engineering, Nanjing University of Science and Technology, Nanjing 210094, China; 2 State Key Laboratory for Diagnosis and Treatment of Infectious Disease, the First Affiliated Hospital, Zhejiang University, Hangzhou 310003, China; 3 School of Chemical Engineering, Nanjing University of Science and Technology, Nanjing 210094, China; 4 Realbio Genomics Institute, Shanghai 200050, China; 5 Kunming Institute of Zoology, Chinese Academy of Science, Kunming 650223, China

## Abstract

Biofilm formation is regarded as one of the major determinants in the prevalence of methicillin-resistant *Staphylococcus aureus* (MRSA) as pathogens of medical device-related infection. However, methicillin-susceptible *S. aureus* (MSSA) can also form biofilm *in vitro* and such biofilms are resistant to vancomycin. Hence, researching the possible mechanisms of MSSA biofilm formation is urgent and necessary. Here, we used *S. aureus* ATCC25923 as the model strain, and studied gene expression profiles in biofilms after the treatment of ursolic acid and resveratrol using RNA-seq technology. The results showed that only ursolic acid could inhibit biofilm formation, which differed from their applied on the multiple clinical drugs resistant MRSA biofilm. RNA-seq data was validated by examining the expression of six genes involved in biofilm formation by qRT-PCR. These data analysis indicated that the mechanism of the MSSA biofilm formation was different from that of the MRSA, due to absence of accessory gene regulator (*agr*) function. These findings suggest that biofilms of *S. aureus* with *agr* dysfunction may be more resistant than those with *agr* function. Therefore, the infection from clinical MSSA may be recalcitrant once forming biofilm. Further study is necessary to uncover the mechanisms of biofilm formation in other clinical *S. aureus.*

Staphylococci are important nosocomial pathogens causing the common hospital and community acquired infective diseases^1^,^2^. The infection is difficult to treat due to their ability to form biofilms^3^. They have an array of virulence factors, including surface proteins responsible to adhesion and invasion of host tissue^4^. Almost all *S. aureus* strains are resistant to penicillin, many of which are resistant to methicillin-related drugs (hereby called Methicillin-resistant *Staphylococcus aureus*, abbr. MRSA strains) and have biofilm-forming capability^1^,^5^,^6^. Traditional antibiotic therapy could only eliminate planktonic cells, leave the sessile forms to survive within the biofilm and continue to disseminate when therapy is terminated^7^. There is evidence that quorum sensing is important for the construction and/or the dissolution of biofilm in some species^8^.

Expression of most virulence factors in *S. aureus* is controlled by the accessory gene regulator locus (*agr*), which encodes a two-component signaling pathway with an active ligand that is a bacterial density sensing peptide (autoinducing peptide [AIP]). The *agr* locus consists of four genes *agr*BDCA^9^. A polymorphism in the amino acid sequence of the auto-inducing peptide and of its corresponding receptor (AgrC) divides *S. aureus* strains into four major groups, *agr*-I, *agr*-II, *agr*-III, and *agr*-IV^10^. All *agr*-II and *agr*-III isolates are defective in *agr*DCA and consequently they do not have a functional AIP as revealed by the lack of transcription of the *hla* gene and the late transcription of δ-toxin^11^. In addition, *agr*-III strains are able to transcribe *ica*R, *sar*A and *rsb*U at the late-exponential and/or the stationary phase. *agr*-I and *agr*-IV variants have functional *agr*-locus, and *sar*A is always expressed, whereas *rsb*U and *ica*R were not transcribed in the mid-exponential and/or the stationary phases. Recent reports indicated that all glycopeptides intermediate *S. aureus* strains (GISA) so far examined belonging to *agr*-II group were defective on *agr* function. Interestingly, these strains were strong biofilm builders^12^,^13^. In this work, we confirmed that *S. aureus* ATCC25923, an *agr*-III strain which is a quality control strain in antimicrobial susceptibility test, is capable to form biofilm *in vitro*. These findings led to the hypothesis that *S. aureus* may developed its ability to form a thick biofilm due to *agr*-locus inactivation, which does not correlate to the antibiotic resistance. Therefore, we chose *S. aureus* ATCC25923 as the model strain to study the possible mechanisms of methicillin-susceptible *S. aureus* (MSSA) biofilm formation.

Studies on global *S. aureus* biofilm transcriptional profiles using microarrays suggested that planktonic cells and biofilm showed distinct patterns of gene expression^14^,^15^,^16^. However, few microarray studies have explored the *S. aureus* biofilms treated with drugs at the genetic level^17^, let alone using the latest RNA-seq technology. Our previous study^18^ showed that ursolic acid inhibited MRSA biofilm formation but had no impact on established biofilm, whereas resveratrol inhibited MRSA biofilm formation and partially removed established biofilm. Therefore, to investigate the potential mechanisms of MSSA biofilm formation at the genetic level, we used high-throughout Illumina sequencing of cDNA (Illumina RNA-seq) to study the differentially expressed genes of *S. aureus* ATCC25923 in biofilm that was treated with ursolic acid and resveratrol, respectively. And then a few of genes related to *S. aureus* biofilm formation were performed qRT-PCR assays to validate RNA-seq results. Finally, these results were compared with the genes expression of MRSA in biofilms under the same conditions^18^.

## Results

### MIC and MBC Determination

The susceptibility of the MSSA planktonic cells to ursolic acid was determined *in vitro* by methods recommended by the Clinical and Laboratory Standards Institute (CLSI). The results showed that the MIC of ursolic acid against *S. aureus* ATCC25923 was 60 μg/mL. In addition, for the MBC of ursolic acid against *S. aureus* ATCC25923, even when saturated in the TSB medium at the final concentration of 200 μg/mL, there were more than five colonies in the plates. So, we concluded that the MBC of ursolic acid against the MSSA was larger than 200 μg/mL.

### Ursolic acid could inhibit MSSA biofilm formation but resveratrol not

**Figure 1:**
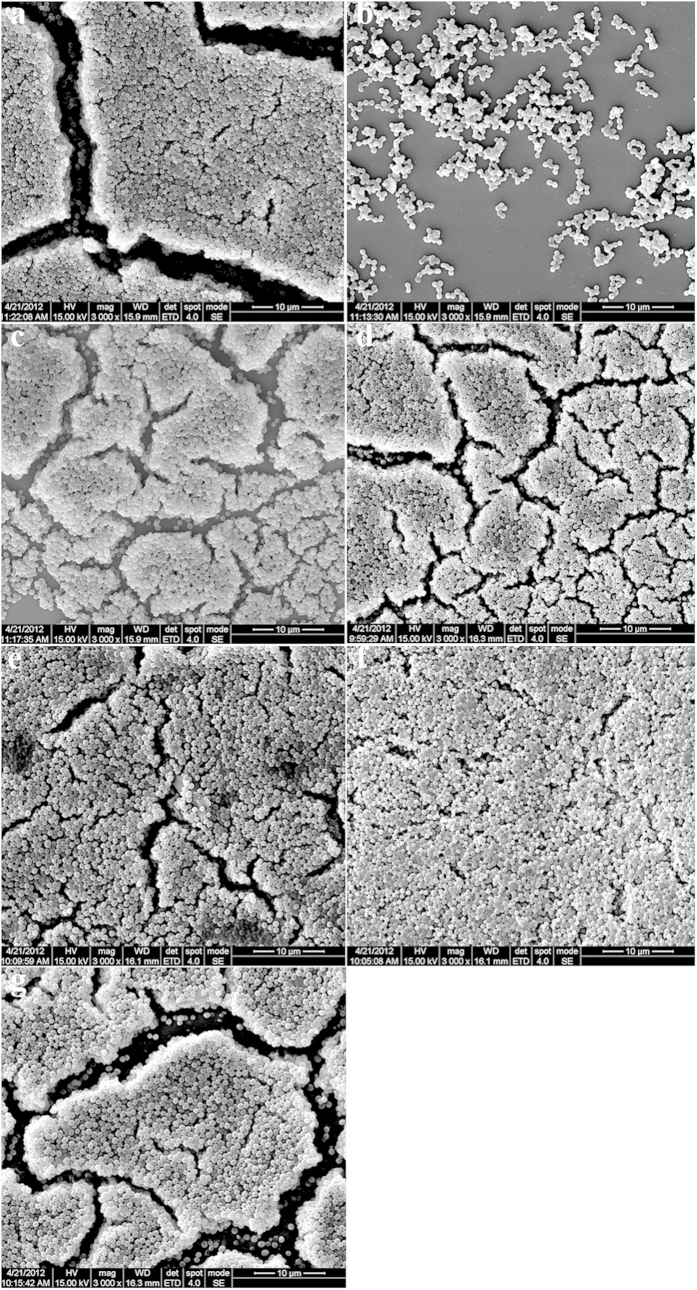
Scanning electron microscopic images showing the structure of the biofilm of *Staphylococcus aureus* ATCC25923. Magnifications, ×3000. (**a**) Control without ethanol (18-h incubation) (218), (**b**) 30 μg/mL ursolic acid (2U30), (**c**) 100 μg/mL resveratrol (2R100), (**d**) control without ethanol (36 h) (236), (**e**) 150 μg/mL resveratrol (2R150), (**f**) 8 μg/mL vancomycin (2V), and (**g**) the mixture of 8 μg/mL vancomycin and 150 μg/mL resveratrol.

The MSSA was able to form biofilms on 96-well plates after 18 h incubation. The SEM images showed that the MSSA formed thick, heterogenous clumps on the coverslips (Fig. 1a,d), which was thicker than MRSA biofilm in the same conditions^18^. Crystal violet staining assays revealed that ursolic acid could inhibit MSSA biofilm formation with an inhibitory rate of 46.50% (Supplementary Fig. S1 online). Moreover, the SEM image showed ursolic acid could inhibit the MSSA biofilm formation (Fig. 1b), while resveratrol could not inhibit the MSSA biofilm formation (Fig. 1c, Supplementary Fig. S1 online).

### Resveratrol, vancomycin, and their mixture could not remove established MSSA biofilm

*In vitro* the effects of resveratrol, vancomycin, and their mixture on established MSSA biofilm were also investigated using the same methods described above with certain modification. Both the semi-quantitative assays and SEM images demonstrated that they had no effect on established MSSA biofilm (Fig. 1e–g, Supplementary Fig. S1 online), which was also different from the fact that they could remove partial established MRSA biofilm^18^.

### Transcriptome assembly and annotation

The cDNA libraries of nine samples (Supplementary Table S1 online) were pooled and subjected to high-throughout sequencing. The generated sequencing reads were used to assemble the transcriptome of *S. aureus* ATCC25923. Two independent Illumina sequencing runs generated a total of 394,463,176 reads consisting of 39,446,317,600 nucleotides (nt) after trimming the adaptors and removing low-quality sequences, with an average length of 100 bp for each short read. Due to the absence of a proper reference genome, Trinity *de novo* assembly^19^,^20^ was applied to construct transcripts. 3,054 transcripts were constructed (Supplementary Fig. S2a online). The length of these assembled transcripts ranged from 200 bp to over 3000 bp, with an average of 850.37 bp (Table 1, Supplementary Fig. S2a online). In total, 3819 coding sequences (CDSs) were generated by TransDecoder (http://transdecoder.sourceforge.net/) and BLAST (Table 1, Supplementary Fig. S2b online). This Transcriptome Shotgun Assembly project has been deposited at DDBJ/EMBL/GenBank under the accession GBKB00000000. The version described in this paper is the first version, GBKB01000000.

Out of the obtained 3819 CDSs, 3807 (99.69%) matched to known protein sequences in the Nr database (Table 2). In addition, the statistical analysis showed that 2341 CDSs (61.5%) had an E-value < 1e-45 (Supplementary Fig. S3a online) and 94.9% of CDSs had alignment identities greater than 95% (Supplementary Fig. S3b online). At a cut-off E-value of 1e-5, 1155 CDSs (30.3%) had matched to the *Staphylococcus aureus* protein database, 836 (22.0%) to the *Staphylococcus aureus* subsp. *aureus* MRSA252 protein database, 298 (7.8%) to the *Staphylococcus aureus* subsp. *aureus* Mu50 protein database and 297 (7.8%) to *Staphylococcus aureus* subsp. *aureus* TCH60 protein database (Supplementary Fig. S3c online).

Gene Ontology (GO) terms were subsequently assigned to *S. aureus* ATCC25923 CDSs based on their sequence matched to known protein sequences in the Nr database. A total of 2876 CDSs (75.31%) and 2074 (67.91%) transcripts were assigned (Table 2). 1942 (63.59%) transcripts were in the biological process category, 1326 (43.42%) transcripts were in the cellular component category, and 1715 (56.16%) transcripts were in the molecular function category (Supplementary Fig. S4 online). In addition, to further elucidate the functionality of *S. aureus* ATCC25923 transcriptome, the annotated CDSs were categorized into different functional groups based on the COG database (Cluster of Orthologus Groups). Out of the 3819 CDSs, 2747 (71.93%) could be classified into 25 COG categories (Table 2). Among the latter, 466 (16.96%) were assigned to the COG category of general function prediction, which was the largest functional group, followed by amino acid transport and metabolism (449, 16.35%), inorganic ion transport and metabolism (354, 12.89%), carbohydrate transport and metabolism (276, 10.05%) and unknown function (273, 9.94%) (Supplementary Fig. S5 online).

### General changes in gene expression after treatment

To determine the gene expression variations between treatment groups and control groups, RNA samples from cells under each condition were sequenced. These nine sample libraries contained clean-up reads ranging from 12,524,649 to 31,001,659. The number of reads from these nine samples that could be mapped to our transcriptome data was then calculated and normalized.

In the study, 130 and 34 transcripts were categorized as up- and down-regulated in response to ursolic acid treatment, respectively. Among these genes, 69 were annotated with known functions with gene names associated with *S. aureus* in the Swiss-Prot database (Supplementary Table S2 online). Although resveratrol had no effect on MSSA biofilm, 407 and 403 transcripts were categorized as significantly regulated under it inhibiting biofilm formation (2R100) and removing established biofilm (2R150) conditions, respectively. In addition, among these transcripts of these two samples, 154 and 190 were annotated with gene names associated with *S. aureus* in the Swiss-Prot database, respectively (Supplementary Table S3 online, Supplementary Table S4 online).

### Differentially expressed genes related to biofilm formation

**Figure 2:**
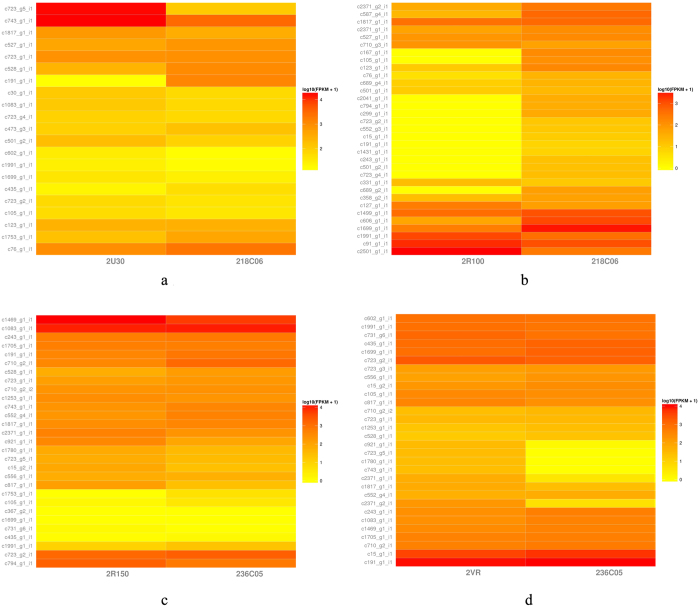
Heatmap of differentially expressed genes associated with *S. aureus* ATCC25923 biofilm formation and virulence. (**a**) ursolic acid inhibiting *S. aureus* ATCC25923 biofilm formation condition, (**b**) resveratrol inhibiting *S. aureus* ATCC25923 biofilm formation condition, (**c**) resveratrol removing established *S. aureus* ATCC25923 biofilm condition, and (**d**) the mixture of resveratrol and vancomycin removing established *S. aureus* ATCC25923 biofilm condition.

Notably, the sets of genes were highly up-regulated and down-regulated in both conditions including some key known genes encoding virulence factors, surface proteins, proteases, and adhesins, which are related to *S. aureus* biofilm formation. In the sample that ursolic acid was used to inhibit MSSA biofilm formation (2U30), 69 detected transcripts showed high similarity to known *S. aureus* genes, many of which were known to be associated with *S. aureus* biofilm formation (Supplementary Table S2 online, Fig. 2a). *ica*R, inhibiting *ica*-dependent biofilm formation in *S. aureus*, was up-regulated by 4.6-fold compared to control. The expressions of genes (*isa*A, *isa*B, *clp*P, and *clp*X) encoding proteases IsaA, IsaB, ClpP, and ClpX increased by 4.4, 2.9, 2.8, and 2.3-fold, respectively. The expression of *sar*X, a *sar*A paralog, increased by 9.5-fold. Moreover, treatment of ursolic acid increased the expression levels of genes that encode adhesins and surface proteins (*fib*, *map*, *sdr*C, *sdr*D, and *spa*) by 2.3, 13.2, 10.7, 5.2, and 5.4-fold, respectively, when MSSA biofilm formation was inhibited. However, the expression of *hld* gene encoding δ-hemolysin, a surfactant and an important element of *S. aureus agr* system, decreased 2.6-fold, which was different from the observation in MRSA biofilm of which ursolic acid is also inhibitive^18^.

Although resveratrol did not inhibit MSSA biofilm formation, the expression levels of many genes related to *S. aureus* biofilm formation changed. Particularly, the expression of *ica*R increased 33.8-fold. In addition, the expressions of *fib*, *isd*C, *isd*I, *map*, *sdr*C, and *sdr*D that encode adhesion and surface protein increased by 2.7, 9.5, 2.0, 7.9, 12.9, and 2.6-fold, respectively. However, the expressions of *ebh* and *fnb*A decreased by 28.8 and 560.3-fold, respectively. Surprisingly, the expressions of the virulence genes (*clp*C, *hla*, *hlb*, *hld*, *hlg*C, *lip*2, and *sar*S) reduced by 2.8, 4.4, 3.5, 6.4, 3.9, 3.3, and 2.5-fold, respectively (Supplementary Table S3 online, Fig. 2b). In spite of the expression levels of these genes changed, resveratrol had no observable impact on MSSA biofilm formation, which was different from the case of applying resveratrol to MRSA biofilm^18^.

When MSSA biofilm was established, both resveratrol and the mixture with vancomycin had no effect, which was different from their effects on established MRSA bioflm^18^. The differentially expressed genes analysis revealed that expressions of *agr*A and *hld* increased by 128.0 and 72.0-fold, respectively, when resveratrol was applied to established MSSA biofilm (2R150). In addition, when the mixture of resveratrol and vancomycin was applied on established MSSA biofilm (2VR), the expressions of *agr*A and *hld* increased by 142.0 and 136.2-fold, respectively. However, when resveratrol was used alone and in the combination with vancomycin on established MRSA biofilm samples, the expression levels of *agr*A and *hld* decreased, which were confirmed by qRT-PCR assays^18^. The expressions of genes (*bbp*, *ebhA*, *map*, *sdr*C, *sdr*D, and *spa*) encoding adhesins and surface proteins in *S. aureus* biofilm formation were up-regulated. Moreover, the expressions of most *cap* locus encoding the capsule polysaccharide synthesis enzymes were not significantly changed, except that *cap*5A increased a little. In the samples 2R150 and 2VR, the expression levels of 21 and 13 genes encoding ribosomal proteins were down-regulated, respectively, which were different from their effects on established MRSA biofilm^18^. It is surprising that the expressions of *bla*Z and *pbp* associated with Beta-lactam-inducible penicillin-binding protein increased thousands folds in the samples 2R150 and 2VR (Supplementary Table S4 online, Supplementary Table S5 online, Fig. 2c–d).

### *S. aureus* ATCC25923 was identified as *agr*-III isolate by polymerase chain reaction (PCR) assays

**Figure 3:**
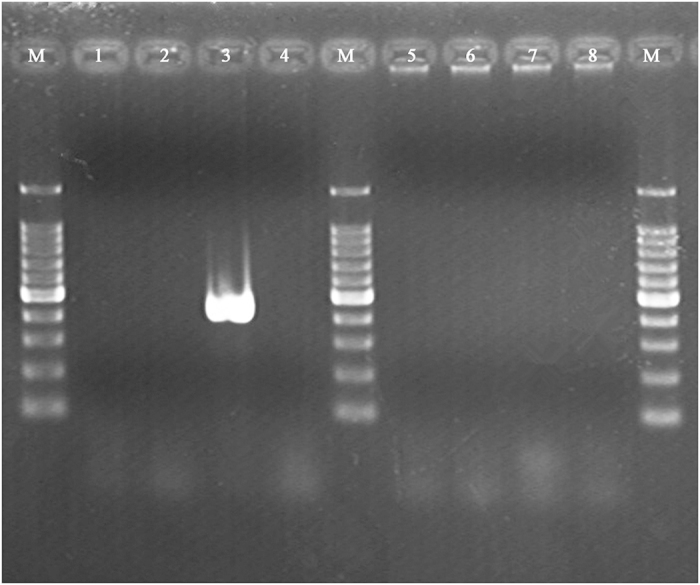
PCR product agarose gel electrophoresis for the identification of *S. aureus* ATCC25923 *agr* genotype. Whole- cell PCR was performed with each of the four *agr* specificity primers. Lines 1 to 4, PCR product using the same forward primer pan-*agr* and different reverse primers (*agr*I, *agr*II, *agr*III, and *agr*IV) with *S. aureus* ATCC25923 genomic DNA as templates (439 bp for *agr*I, 573 bp for *agr*II, 406 bp for *agr*III, and 657 bp for *agr*IV); Lines 5 to 8, negative controls responsible to lines 1 to 4, respectively; Lines M, 100 bp for DNA ladder.

*S. aureus* ATCC25923 *agr* specificity was identified by the expected PCR product size according to Shopsin *et al.* report^21^. The lengths of the PCR products were estimated by comparing with the 100 bp DNA ladder (Takara, Japan). The results showed that only *agr*-III genotype was obtained by the PCR assay and the length of the PCR product was 406 bp, which corresponded to expected product size (Fig. 3). In addition, we also sequenced the PCR product and aligned it with *Staphylococcus aureus* RN8462 (GenBank accession number AF001783) using BLAST, which is *agr* group III strain^10^,^22^,^23^. The results revealed that their identity was 99% and amplified fragment corresponding to the region was right. Therefore, based on above results, we determined that *S. aureus* ATCC25923 was *agr*-III genotype strain.

### RNA-seq results were verified by qRT-PCR

**Figure 4:**
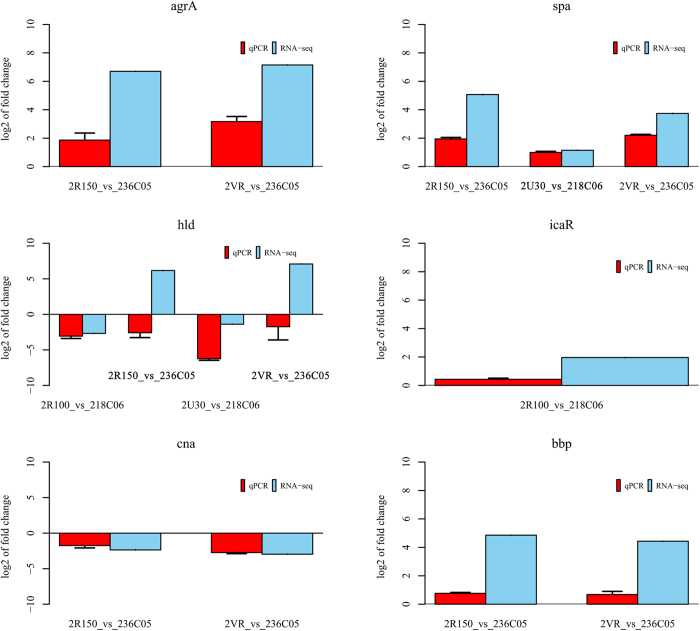
Expression ratio (log_2_) obtained by qRT-PCR and RNA-seq of these six selected genes associated with *S. aureus* biofilm formation. *pyk* was used as a reference gene for normalization of qRT-PCR data. All gene expression ratios from qRT-PCR between treatment groups and controls under different conditions were significantly different (P < 0.05). Bars represent the error standard (n = 3). The x-axis indicates the comparison between treatment groups and controls under different conditions. The y-axis shows the gene expression ratios.

On the basis of these six genes (*agr*A, *hld*, *ica*R, *spa*, *cna*, and *bbp*) were significantly differential transcribed (P < 0.05, FDR < 0.001) under inhibiting biofilm formation condition or removing established biofilm condition and their expression was related to *S. aureus* biofilm formation, they were selected for qRT-PCR to investigate gene expression difference between treatment samples and controls. The results of gene expression ratios between treatment groups and controls are shown in Fig. 4. Although the trend of *hld* expression ratio under removing established biofilm condition was inconsistent between RNA-seq and qRT-PCR, a liner regression analysis showed an overall correlation of *r *= 0.842 for these six genes under inhibiting biofilm formation condition or removing established biofilm condition, which indicates a high correlation between transcript abundance assayed by qRT-PCR and the transcription profile revealed by RNA-seq data^24^. In general, although the exact fold difference for each gene by qRT-PCR was different from the RNA-seq, the comparison pairs had the similar trends with RNA-seq, which suggested that there would be the relative high consistency between RNA-seq and qRT-PCR.

## Discussion

*S. aureus* ATCC25923, a quality control strain in antimicrobial susceptibility test, is a clinical isolate and able to form biofilm *in vitro*^25^. Vancomycin had no effect on its biofilm formation (Supplementary Fig. S1 online), indicating that the formation of bacterial biofilm may be the first, and probably an important barrier of multiple drugs resistance. Therefore, we employed *S. aureus* ATCC25923 as the model strain to study the mechanisms of MSSA biofilm formation. Qin *et al.*^18^ reported that ursolic acid could inhibit MRSA biofilm formation, and resveratrol could not only inhibit MRSA biofilm formation but also remove partial established MRSA biofilm. However, only ursolic acid could inhibit *S. aureus* ATCC25923 biofilm formation. Given above differences, the processes of MRSA and MSSA biofilm formation may be different. So, we studied the gene expression profiles of *S. aureus* ATCC25923 in bioflms after the treatment of ursolic acid and resveratrol using RNA-seq technology.

In this work, with the RNA-seq technology a total of 3054 transcripts were obtained, representing a comprehensive transcriptome of *S. aureus*. Functional annotation showed that these transcripts covered every basic biological process. Moreover, we used Trinity *de novo* assembly to connect the short reads, which is believed to be more suitable for constructing a *de novo* transcriptome without a reference genome than other *de novo* assemblers of RNA-seq^19^,^20^. In addition, the assembled transcripts were matched with genome sequences from *Staphylococcus aureus* subsp. *aureus* NCTC 8325: 47.70% of matched sequences had an E-value lower than 1e-20. We also aligned reads to evaluate the integrity and reliability of the assembly. The results showed that 99.4% of reads mapped the assembled transcripts, which confirmed that the *de novo* assembly we performed was accurate.

Intercellular signaling, often referred to quorum sensing, has been shown to involve in biofilm development in several bacteria^8^. The *S. aureus* quorum sensing system is encoded by the *agr* locus, which consists of four genes *agr*BDCA^9^. Although structurally conserved, *agr*B, D and C have diverged widely among Staphylococci, giving rise to different specificity groups^11^,^21^. *S. aureus* ATCC25923 was classified as *agr*-III strain as mentioned by others^26^,^27^. In this research, *agr*D was not found in the global transcriptome, but *ag*rA, *ica*R, *hld*, *sar*A, and *spa* were detected. In addition, we validated the results from RNA-seq analysis by PCR and qRT-PCR assays. Our results confirmed that *S. aureus* ATCC25923 is indeed an *agr*-III strain, namely the *agr* system of this strain is nonfunctional. Due to the differential expression of *ica*R found in transcriptome, we speculated that the biofilm formation may be *ica*-dependent, which was validated by qRT-PCR assay.

In this study, we also performed comparative analysis between different control samples (218 & 236). Both crystal violet staining assays and SEM images revealed that *S. aureus* ATCC25923 biofilm formed at 18 h was similar to the biofilm formed at 36 h (Fig. 1, Supplementary Fig. S1 online). Moreover, gene expression analysis showed that *fib*, *spa*, *sar*A, and *sar*X were down-regulated in the sample 236 compared to the sample 218. However, the expression levels *clp*B, *clp*C, *hlb*, *hlg*A, *hlg*B, *lip*1, *lip*2, and *sbi* that encode virulence factors increased by 7.4, 24.8, 3.2, 4.0, 4.7, 2.4, 2.5, and 2.1-folds, respectively. These three aspects showed that *S. aureus* ATCC25923 could form stable and mature biofilm after 18 h incubation. Meanwhile, *S. aureus* ATCC25923 is *agr*-III strain, which formed thicker biofilm than the MRSA strain. The MRSA biofilm previously described by our group was thicker after 36 h than that formed after 18 h^18^. RNA-seq data analysis showed that in the sample M36, *spl*A, *spl*B, *spl*C, and *ssp*B2 genes that encode extracellular proteases and are controlled by the *agr* system^27^ were significantly down-regulated compared to the sample M18, indicating that the *agr* is active in the MRSA strain. Through comparative analysis, we confirmed that i) the MSSA biofilm developed at a quicker rate than MRSA, ii) the MSSA biofilm was thicker than MRSA biofilm due to the absence of *agr* function, and iii) the ability of *S. aureus* to form biofilm is independent on antibiotic resistance. Therefore, it is not difficult to understand that i) resveratrol did not inhibit MSSA biofilm formation, and ii) resveratrol, vancomycin and their mixture had no effect on established MSSA biofilm. Meanwhile, Qin *et al.*^18^ proposed that resveratrol inhibited MRSA biofilm formation by disturbing the QS system. Based on the comparative analysis of resveratrol effecting on *S. aureus* biofilm in different *agr* genotypes, we suggest that resveratrol would be used to block *S. aureus* biofilm formation in which the *agr* system is active.

In conclusion, RNA-seq data analysis in this study suggested that the mechanism of biofilm formation in clinical MSSA could be different from that of the clinical MRSA. *S. aureus* may have an enhanced ability to form a thick biofilm if the *agr*-locus is inactivated, and their resistance to antibiotics does not depend on the functionality of the *agr* system. In addition, we speculated that biofilm formed by MSSA strains may be more resistant than the biofilm of MRSA strains, which was proved by Bauer *et al.* using other approaches^28^. Therefore, the infection from clinical MSSA may be more recalcitrant once forming biofilm. Further study is necessary to uncover the mechanisms of biofilm formation in other clinical *S. aureus* isolates.

## Methods

### Strain and growth conditions

*S. aureus* ATCC25923 was provided by the Clinical Laboratory Department of the First Affiliated Hospital, Nanjing Medical University, Nanjing, China. The strain was susceptible to many antibiotics determined by broth dilution method as recommended by the Clinical and Laboratory Standards Institute (CLSI, M100-S22), and their MICs were shown in Supplementary Table S6. In addition, *S. aureus* ATCC25923 was identified as methicillin-susceptible strain using cefoxitin disk diffusion and oxacillin broth dilution methods as recommended by CLSI (M100-S22). Blood agar medium was used to culture the bacterium. Nutrient broth (NB) medium was used for routine cultivation, while trypticase soy broth (TSB) medium was used to study the effects of natural compounds on biofilms in 96-well flat-bottom polystyrene plates (Costar 3599; Corning; USA). Resveratrol and ursolic acid used in this study were isolated from natural products by our group and their purities were confirmed to be >98% using high performance liquid chromatography methods. They were dissolved in ethanol at 30 mg/mL and 5 mg/mL, respectively. The vancomycin was purchased from Sigma and dissolved in water at 5 mg/mL. All compounds were filtered with a 0.22-μm filter in sterile conditions and then stored at 4 °C.

### Determination of Minimal inhibitory concentration (MIC) and minimal bactericidal concentration (MBC)

MIC and MBC were determined by a microtitre broth dilution method as recommended by the Clinical and Laboratory Standards Institute (CLSI) with a little modification. Briefly, the test medium was TSB and the density of bacteria was 5 × 10^5^ colony forming units (CFU)/mL. Cell suspensions (200 μL) were inoculated into the wells supplemented with ursolic acid at different concentrations (30, 40, 50, 60, 70, 80, and 90 μg/mL). These concentrations were selected based on the fact that the solubility of ursolic acid in TSB medium decreases as its concentration increases. We did not measure the MIC and MBC of resveratrol because it did not inhibit MSSA biofilm formation. The inoculated microplates were incubated at 37 °C for 18 h and examined. MIC was defined as the lowest concentration of the drug that inhibited >90% of the growth of the test microorganism^29^.

The MBC was obtained by subculturing 100 μL from each well from the MIC assay onto TSA plates. The plates were incubated at 37 °C for 24`h and MBC was defined as the lowest concentration of substance when the subcultures developed no more than five colonies on each plate^7^. Three replicates were carried out in the experiment.

### Inhibition of resveratrol and ursolic acid on MSSA biofilm formation

This assay was performed as previously described by our group with some modification^18^. Briefly, MSSA culture was supplemented with resveratrol or ursolic acid at final concentrations of 100 μg/mL and 30 μg/mL, respectively, because both of concentrations were previously applied on MRSA biofilm by our group^18^. The plates were incubated at 37 ^o^C for 18 h with shaking at 150 rpm. The absorbance at OD_570 nm_ was measured with a microplate reader (BioTek, USA). All assays were performed with independent triplicates. The inhibition rates were calculated using previously described formula^18^.

### Treatment of MSSA biofilm with resveratrol, vancomycin, or their mixture

These assays were performed as previously described by our group with a slight modification^18^. In brief, a total of 200 μL of fresh TSB with resveratrol (final concentration, 150 μg/mL), vancomycin (final concentration, 8 μg/mL), or their mixture (resveratrol 150 μg/mL + vancomycin 8 μg/mL) was added to the wells with established MSSA biofilm. The plates were incubated at 37 ^o^C for 18 h with shaking. All assays were performed with independent triplicates.

### Scanning electron microscope (SEM) measurement assays

SEM was performed with a scanning electron microscope (FEI Quanta 200; USA) on biofilm formed on glass coverslips according to methods described by Qin *et al.* with a little modification^18^. The observations were usually performed.

### Total RNA isolation

All samples used for the RNA sequencing were prepared in the 24-well flat bottomed polystyrene plates. After the biofilms were rinsed with sterile water, the biofilm cells were scraped with pipette and suspended in the RNAprotect Bacteria Reagent (Qiagen, Germany). The cell suspension was then transferred to a microcentrifuge tube and incubated for 5 min at room temperature to stabilize the mRNA. Next, the cell suspensions were centrifuged at 8,000 × *g* for 5 min to pellet the cells. Total RNA was purified from the pellet using RNeasy Mini Kit (Qiagen) according to the manufacturer’s protocol with modification described by Qin *et al.*^18^. Each RNA sample was suspended in 30 μL of RNA storage solution and the quality of total RNA obtained was determined using Agilent 2100 Bioanalyzer.

### Enrichment and sequencing of mRNA

A total of 10 μg of each RNA sample was subjected to further purification using a MICROBExpress Kit (Ambion, USA) according to the manufacturer’s protocol. The mRNA sample was suspended in 25 μL RNA storage solution and the quality of mRNA was determined using Agilent 2100 Bioanalyzer. Bacterial mRNA was fragmented using a RNA fragmentation kit (Ambion), and the fragments were achieved in the size range of 200–250 bp. Double-stranded cDNA was generated using the SuperScript Double-Stranded cDNA Synthesis Kit (Invitrogen, Carlsbad, CA). An Illumina Paired End Sample Prep kit was used to prepare RNA-seq library. All of the samples were sequenced using the Hiseq2000 (Illumina, CA) sequencer at Beijing Genomics Institute at Shenzhen.

### Transcriptome assembly and annotation

Due to lack of the reference genome of *S. aureus* ATCC25923, we used Trinity *de novo* assembler (version 20140413)^19^,^20^ to assemble these RNA-seq sequences. The raw reads were filtered using NGS QC ToolKit software^30^. The low-quality reads (which contained adaptor contamination, the pair-end reads satisfied N > 10%, and low quality reads (quality value < 20)>50%) were removed.

Coding sequences (CDSs) were identified using TransDecoder (http://transdecoder.sourceforge.net/). These CDSs were used for BLAST searches against the NCBI Nr protein database (NCBI non-redundant sequence database) (version 2014-5) with an E-Value cut-off of 1e-5. CDSs were further aligned by BLASTX to protein database including Swiss-Prot (version 2014-5)^31^, KEGG (version 2013-10)^32^,^33^,^34^, COG (version 20090331)^35^,^36^, and GO (version 2014-5)^37^ to retrieve their functional annotations. If results of different database conflicted, a priority order of Nr, Swiss-Prot, KEGG, COG and GO was followed. For transcripts that were not identified by TransDecoder software but were annotated by above protein databases, they were still regarded as CDSs.

The Blast2GO program^38^ was used to obtain GO annotation for CDSs, as well as for KEGG and COG analyses. The WEGO software^39^ was used to perform GO functional classification. This analysis mapped all of the annotated CDSs to the GO terms in the database and calculated the number of CDSs associated with every GO term. COG and KEGG pathway annotations were performed using Blastall software (version 2.2.25) against the COG and KEGG databases.

### Identification of differentially expressed genes

The RNA-seq reads were mapped to our transcriptome reference database, and transcript abundances were quantified by RSEM^40^. Genes that differentially expressed among these nine samples were identified using the numbers of mapped reads as EdgeR inputs^41^. Genes with an adjusted *P* value ≤ 0.05, FDR ≤ 0.001 and fold change ≥ 2 were identified as being differentially expressed. Differentially expressed genes were regarded as up-regulated if their expression levels in treated samples were significantly higher than those in the control samples. Similarly, they were down-regulated if their expression levels in treated samples were significantly lower than those in the control samples.

### Determined *S. aureus* ATCC 25923 *agr* class by PCR assays

*S. aureus* ATCC25923 genomic DNA was isolated using Ezup Column Bacteria Genomic DNA Purification kit (Sangon Biotech, China) according to the manufacturer’s recommended protocol. The quality of genomic DNA obtained was determined using Agilent 2100 Bioanalyzer and 0.7% agarose gel, which was stained with ethidium bromide (0.5 μg/mL).

Based on analysis of RNA-seq data, we speculated that *S. aureus* ATCC25923 would be *agr*-III isolate. Therefore, to verify the results from RNA-seq analysis, *agr* class-specific primer pairs were designed for *S. aureus* ATCC25923 *agr* genotype measurement according to Shopsin *et al.* report^21^ (Supplementary Table S7 online).

Amplification of *agr* was carried out in a 25 μL volume using 2 × Taq Master Mix (Vazyme, China) according to the manufacturer’s recommended protocol. The reaction was performed using Authorized Thermal Cycler (Eppendorf, Germany) with the following cycle parameters: an initial 5 min denaturation step at 94 ^o^C, followed by 26 cycles of 30 sec at 94 ^o^C, 30 sec at 55 ^o^C, 1 min at 72 ^o^C, and extension for 10 min at 72 ^o^C. After PCR, the product was separated on 2.0% agarose gel, which was stained with ethidium bromide (0.5 μg/mL). All measurements were independently conducted 3 times. The PCR product was recycled using an AxyPrep DNA Gel Extraction kit (Axygen, USA) and then ligated into the pMD19-T vector using pMD^TM^19-T Vector Cloning kit (TakaRa, Japan) according to manufacturer’s instructions. Next, they were transferred into *E. coli* DH5α competent cell and then cultured on LB agar plate supplemented with 100 μg/mL ampicillin overnight at 37 ^o^C. White colonies were grown in LB broth supplemented with 100 μg/mL ampicillin overnight incubation. The plasmid DNA containing the PCR fragment insert was isolated from each culture using AxyPrep Plasmid Miniprep kit (Axygen, USA) according to manufacturer’s instructions. All DNA samples with A_260_/A_280_ above 1.8 were sequenced using 3730XL automated sequencer (Applied Biosystems, USA) at Shanghai Sunny Biotechnology Company, China. All measurements were independently conducted five times. BLAST (http://blast.ncbi.nlm.nih.gov/Blast.cgi) was used to obtain a multiple sequence alignment of sequences from five DNA samples and to identify the region of *agr* locus that was amplified through alignment with *Staphylococcus aureus* strain RN8462 (GenBank accession number AF001783), which is *agr* group III strain^10^,^22^,^23^.

### qRT-PCR assays

Cafiso *et al.*^11^ reported that strains with *agr*-III have an inactive *agr*-system, but were able to transcribe *ica*R, *hld*, and *sar*A at late- and post-exponential phases, which are related to biofilm production. Herein, *S. aureus* ATCC25923 may be *agr*-III isolate and four genes (*agr*A, *hld*, *ica*R, and *spa*) were significantly differential transcribed (P < 0.05, FDR < 0.001) under inhibiting biofilm formation condition or removing established biofilm condition. Therefore, these four genes were selected for qRT-PCR to validate RNA-seq data. Although *sar*A was transcribed in all samples, it was not significantly differential transcribed between all treatment samples and controls. So, *sar*A gene was not selected for qRT-PCR. In addition, under removing established biofilm condition, these two genes (*bbp* and *cna*) also were significantly differential transcribed and their expression was related to *S. aureus* biofilm formation. So, they were also selected for qRT-PCR. In general, these six genes (*agr*A, *hld*, *ica*R, *spa*, *cna*, and *bbp*) were selected for qRT-PCR assays. Total RNA was reverse transcribed into cDNA using the HiScript^®^ Q RT SuperMix for qPCR (+gDNA wiper) (Vazyme, China) according to the manufacturer’s protocol. The resulting cDNAs were stored at −20 ^o^C until they were required. The qRT-PCR was carried out in a 20 μL volume using AceQ^TM^ qPCR SYBR^®^ Green Master Mix (Vazyme, China) as recommended by the manufacturer. These reactions were performed using the Applied Biosystems 7300 Real-time PCR System, and all cycle parameters were shown in Supplementary Table S7. All measurements were done in three independent replicates and additionally four replications within each qRT-PCR run. For data normalization, housekeeping gene *pyk* was used as an internal reference to obtain basis of normalization. Fold change between treatment samples and controls was calculated using 2^–ΔΔCt^ method. All primers and sequences are listed in Supplementary Table S7.

### Statistical analysis

At least three independent replicates of each 96-well plate experiment were performed. The biofilm inhibition results and qRT-PCR results were statistically analyzed using SPSS software version 18.0 (SPSS, Chicago, IL, USA). P values ≤ 0.05 were considered significant. In addition, a liner regression analysis was done to obtain the correlation between transcript abundance assayed by qRT-PCR and transcription profile revealed by RNA-seq data. The correlation coefficient (*r*) was obtained by Pearson’s analysis.

## Additional Information

**How to cite this article**: Tan, X. *et al.* Transcriptome analysis of the biofilm formed by methicillin-susceptible *Staphylococcus aureus*. *Sci. Rep.* **5**, 11997; doi: 10.1038/srep11997 (2015).

## Supplementary Material

Supplementary Information

## Figures and Tables

**Table 1:** Summary of the statistics of RNA-seq and *de novo* assembly of the*Staphylococcus aureus* ATCC25923 project.

	Number	Mean size	N_50_ size	Total Nucleotides
Read	394,463,176	100	100	39,446,317,600
Transcript	3,054	850.37	1,860	2,597,031

**Table 2:** Summary of annotations of the assembled coding sequences (CDSs) and transcripts in *Staphylococcus aureus* ATCC25923.

Category	The number of CDSs	The number of transcripts
Nr	3807	2768
Swiss-prot	2649	1830
KEGG	2553	1780
COG	2747	1871
GO	2876	2074
All	3819	3054
