# Supplementary material for: Transcriptome analysis of the biofilm formed by methicillin-susceptible Staphylococcus aureus
Source: Sci Rep. 2015 Jul 7;5:11997. doi: 10.1038/srep11997 (PMC4493712; doi:10.1038/srep11997)
Supplement: Supplementary Information [file srep11997-s1.pdf]

SUPPLEMENTARY FILE

Transcriptome analysis of the biofilm formed by methicillin-susceptible *Staphylococcus aureus*

Xiaojuan Tan[1§], Nan Qin[2§], Chunyan Wu[4], Jiyang Sheng[1], Rui Yang[1], Beiwen Zheng[2], Zhanshan Ma[5], Lin Liu[2], Xinhua Peng[3]* & Aiqun Jia[1]*

[1] School of Environmental and Biological Engineering, Nanjing University of Science and Technology, Nanjing 210094, China

[2] State Key Laboratory for Diagnosis and Treatment of Infectious Disease, the First Affiliated Hospital, Zhejiang University, Hangzhou 310003, China

[3] School of Chemical Engineering, Nanjing University of Science and Technology, Nanjing 210094, China

[4] Realbio Genomics Institute, Shanghai 200050, China

[5] Kunming Institute of Zoology, Chinese Academy of Science, Kunming 650223, China

[§] These authors contributed equally to this work

* Corresponding author

Correspondence and requests for materials should be addressed to A.J. (email: jiaaiqun@gmail.com) and X.P. (email: xinhuapeng@mail.njust.edu.cn).

Supplementary figures

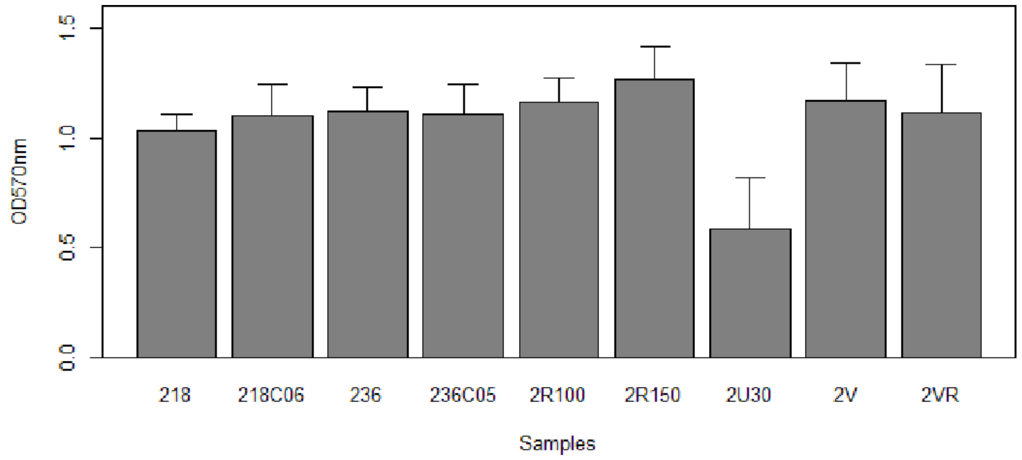

Supplementary Figure S1. Inhibition of *Staphylococcus aureus* ATCC25923 biofilm by ursolic acid, resveratrol, and vancomycin. At least three independent experiments were conducted, and the error bars indicate one standard deviation. The naming of these samples was shown in Supplementary Table S1 online. 218C06 and 236C05 were prepared to observe the effects of solvent on biofilms in both different conditions. Value treated with ursolic acid (2U30) was significantly different from the untreated control (218C06) (Student's t-test). Samples treated with resveratrol, vancomycin and their mixture were not significantly different from the untreated control (Student's t-test). Samples treated with ethanol were not significantly different from the control (Student's t-test).

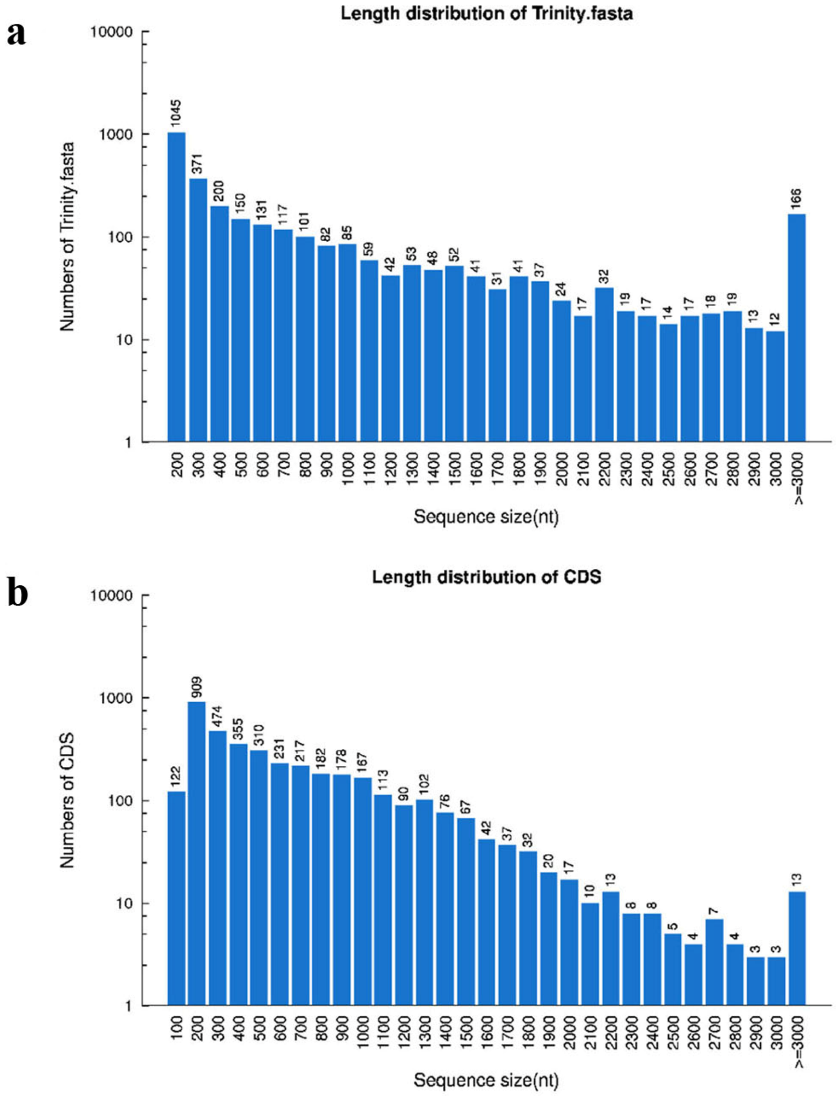

Supplementary Figure S2. Statistical analysis of *de novo* assembly of *Staphylococcus aureus* ATCC25923 short reads generated by Illumina Hiseq2000 sequencing. (**a**) Length distribution of assembled transcripts and (**b**) length distribution of CDS.

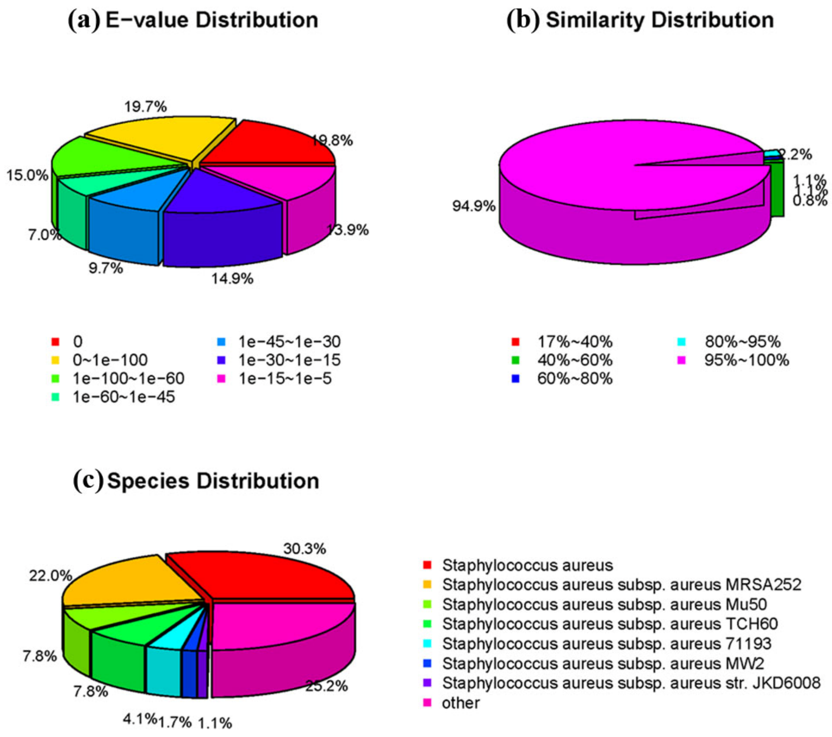

Supplementary Figure S3. Summary of Nr annotation results of *de novo* assembled CDSs in *Staphylococcus aureus* ATCC25923. (**a**) E-value distribution, (**b**) similarity distribution, and (**c**) species distribution.

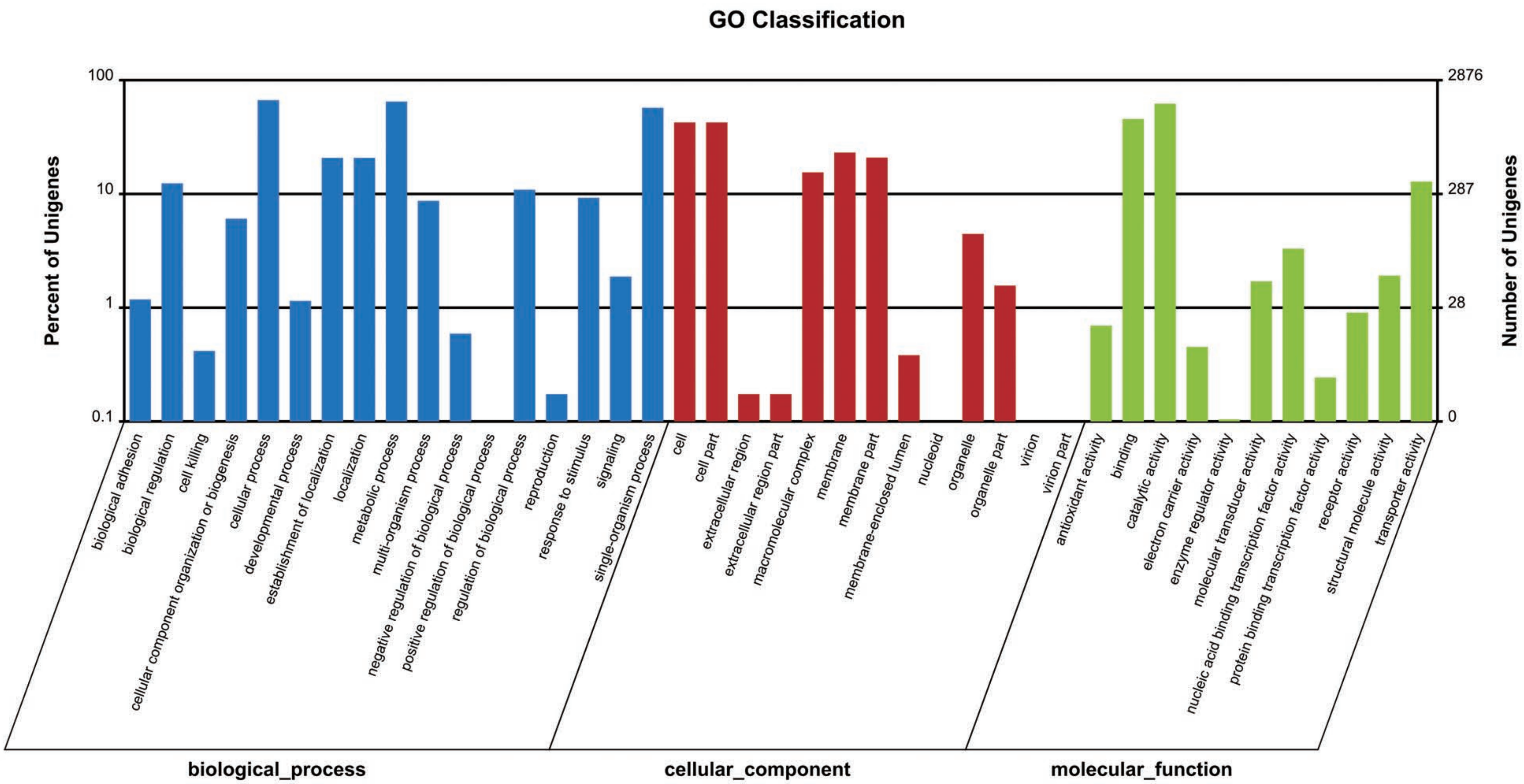

Supplementary Figure S4. Histogram of GO classifications of assembled *Staphylococcus aureus* ATCC25923 CDSs. Results are summarized for three main GO categories: biological process, cellular component and molecular function.

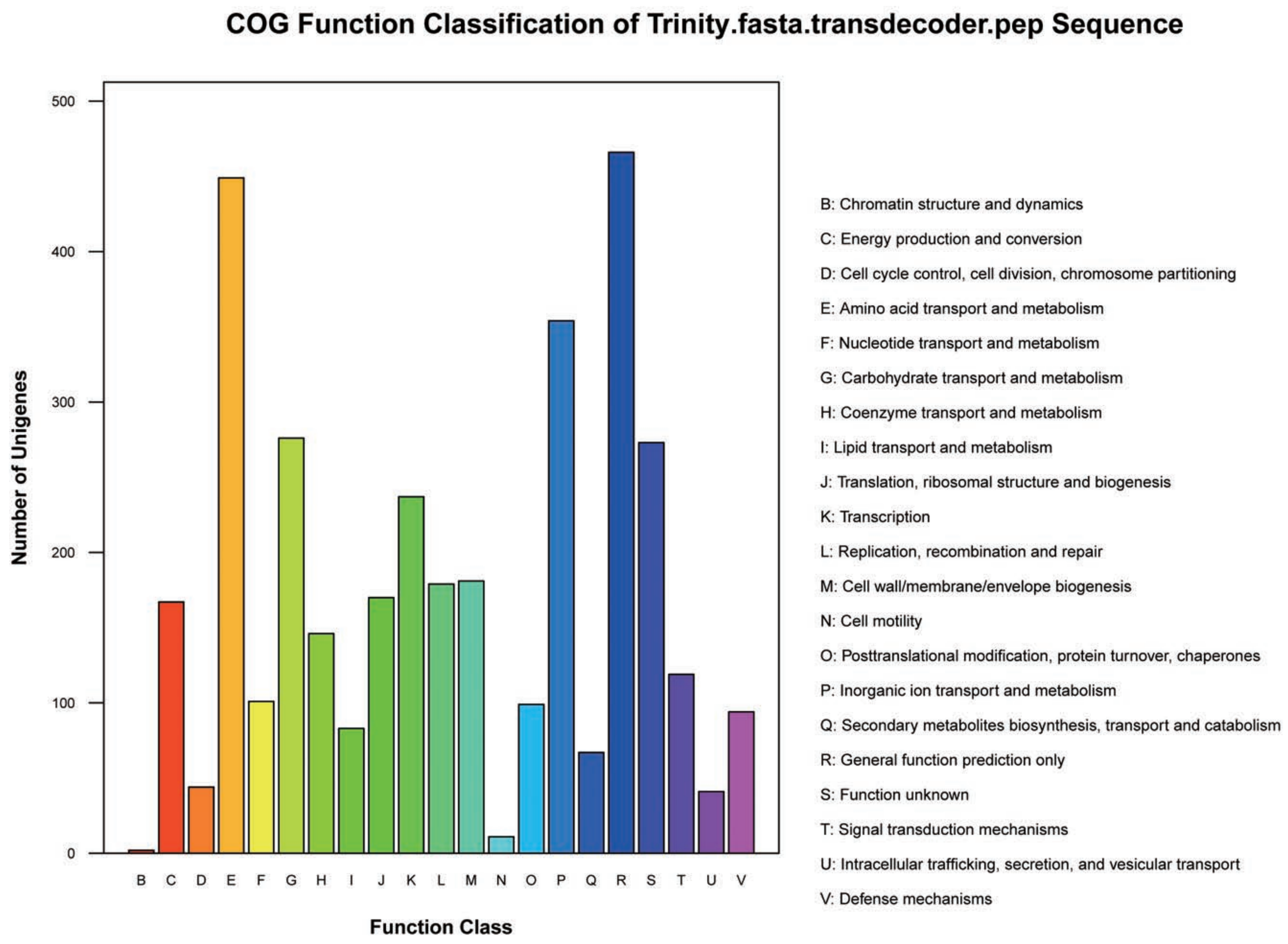

Supplementary Figure S5. Histogram of COG classification of *Staphylococcus aureus* ATCC25923 sequences deposited at the NCBI database and *de novo* assembled CDSs.

Supplementary tables

Supplementary Table S1. The naming of these nine samples in the different conditions

Condition	Compound	Sample name
Inhibiting biofilm formation condition	Control without ethanol	218
	0.6% [v/v] ethanol	218C06
	30 μg/mL Ursolic acid	2U30
	100 μg/mL Resveratrol	2R100
Removing established biofilm condition	Control without ethanol	236
	0.5% [v/v] ethanol	236C05
	150 μg/mL Resveratrol	2R150
	8 μg/mL Vancomycin	2V
	The mixture of 8 μg/mL vancomycin and 150 μg/mL Resveratrol	2VR

Supplementary Table S2. Differentially expressed genes in 2U30 compared with 218C06

ID	log2(fold_change)	logCPM	P_Value	FDR	gene name
c477_g2_i1	-1.08E+00	8.002470992	6.99E-07	1.33E-05	arcAB
c674_g2_i1	-1.135759769	9.75560995	6.44E-08	1.51E-06	arsRBC
c1168_g1_i1	-1.23699429	5.989416591	9.28E-07	1.70E-05	azoR
c555_g1_i1	1.087031876	7.623736979	1.82E-06	3.09E-05	bsaA
c434_g1_i1	-1.480764183	11.83316246	1.50E-12	9.83E-11	cadCA
c343_g1_i1	1.182838368	9.143016369	3.42E-08	8.83E-07	ccpA
c40_g1_i1	1.001872235	10.01512087	1.87E-06	3.14E-05	cidB
c743_g1_i1	1.65429996	12.02136612	4.13E-15	3.76E-13	clpB
c1083_g1_i1	2.603771042	8.740114402	2.86E-29	2.99E-26	clpL
c602_g1_i1	1.506440624	9.860137057	1.69E-12	1.07E-10	clpP
c1699_g1_i1	1.173005899	8.348636596	9.97E-08	2.24E-06	clpX
c731_g5_i1	-1.305321318	7.557888897	4.97E-09	1.60E-07	copZ
c846_g1_i1	1.572292613	5.000000134	1.60E-06	2.79E-05	crcB1
c2044_g1_i1	1.239913211	10.31156131	3.94E-09	1.31E-07	cspA
c1691_g1_i1	1.635325465	11.31321485	9.93E-15	8.65E-13	dltAC
c123_g1_i1	-1.410319235	4.227941008	4.17E-05	0.000456257	ebhA
c105_g1_i1	-1.32872828	6.387505614	4.48E-08	1.10E-06	ebhA
c2020_g1_i1	1.837703467	7.062532226	8.95E-14	6.94E-12	esxA
c1749_g1_i1	1.050321932	10.12102976	5.78E-07	1.13E-05	fda
c1996_g1_i1	1.126181162	9.885622689	9.63E-08	2.19E-06	femBA
c76_g1_i1	1.221790067	5.287888461	5.82E-05	0.00061462	fib
c800_g1_i1	-1.302410843	5.638640286	7.63E-07	1.41E-05	flr
c2386_g1_i1	1.136648046	8.990919753	1.22E-07	2.72E-06	glcT
c220_g1_i1	2.466970295	8.53411047	1.97E-26	8.26E-24	glpD
c594_g1_i1	1.064410721	9.997007498	4.28E-07	8.45E-06	groLS
c731_g2_i8	4.875172262	3.389782975	2.78E-09	9.71E-08	guaAB
c191_g1_i1	-1.394303681	15.33625765	2.12E-11	1.01E-09	hld
c718_g1_i1	1.204452443	11.4708007	7.80E-09	2.44E-07	hup
c501_g2_i1	2.219428503	4.197313622	4.20E-07	8.38E-06	icaR
c1991_g1_i1	2.144688105	9.390941272	2.09E-22	7.29E-20	isaA
c1753_g1_i1	1.538101982	10.26513185	4.55E-13	3.28E-11	isaB
c333_g2_i1	-1.235732674	4.627718557	8.49E-05	0.000862304	lacAB
c474_g4_i1	-1.503049517	5.043340212	2.15E-07	4.51E-06	lacDFE
c474_g2_i1	-1.092744746	6.531077938	4.49E-06	6.85E-05	lacEG
c731_g14_i1	-1.135971891	9.606296852	6.73E-08	1.55E-06	lip1
c54_g1_i1	1.186857614	9.030376487	3.36E-08	8.78E-07	lytH
c30_g1_i1	1.621804486	7.557228491	4.20E-12	2.44E-10	map
c1817_g1_i1	2.10253038	6.550903149	2.30E-15	2.29E-13	map
c1447_g1_i1	1.042951867	10.0151323	7.14E-07	1.35E-05	mprF
c677_g1_i1	1.146252834	10.78406326	4.30E-08	1.07E-06	mutS2
c2121_g1_i1	1.300008727	8.332777779	4.36E-09	1.43E-07	nfrA
c571_g1_i1	1.043342065	7.333515667	6.70E-06	9.74E-05	oatA
c2336_g1_i1	-1.620515217	12.07909123	1.20E-14	1.00E-12	pdxST
c392_g2_i1	1.071791184	6.923591525	8.00E-06	0.000113091	pepA
c735_g1_i1	-1.035082646	6.10779214	2.93E-05	0.000344443	ptsG
c1992_g1_i1	1.3691562	6.503718305	6.52E-08	1.52E-06	purA
c2100_g1_i1	1.012061232	8.670534896	2.83E-06	4.51E-05	purB
c394_g1_i1	1.933955133	10.65726542	1.88E-19	3.57E-17	purQLMEDCNH
c1477_g1_i1	1.150866182	6.435723187	5.60E-06	8.31E-05	pyrB
c642_g1_i1	1.478293402	8.574418139	2.09E-11	1.01E-09	ribU
c117_g2_i1	1.491038668	7.991755291	4.54E-11	2.11E-09	rpmE2

c225_g1_i1	1.301571045	8.361886849	3.96E-09	1.31E-07	rpsD
c46_g1_i1	1.636723353	7.977094177	7.44E-13	5.02E-11	rpsT
c1378_g1_i1	1.022454442	10.37801409	1.05E-06	1.90E-05	rsmH
c473_g3_i1	3.246826959	7.566178989	2.72E-35	5.70E-32	sarX
c527_g1_i1	-1.250854321	7.15090512	4.17E-08	1.05E-06	sasA
c723_g4_i1	1.311795847	5.25183535	1.73E-05	0.000223198	sdrC
c723_g2_i1	2.107476875	6.59681436	1.66E-15	1.74E-13	sdrC
c723_g5_i1	1.045681935	6.471417625	3.02E-05	0.000352448	sdrD
c723_g1_i1	1.316031667	5.887555847	1.76E-06	3.02E-05	sdrD
c662_g1_i1	1.021719783	7.597242559	7.08E-06	0.000102125	serS
c435_g1_i1	1.147950328	7.361597819	7.49E-07	1.40E-05	spa
c528_g1_i1	1.279368761	7.993367861	1.22E-08	3.66E-07	spa
c720_g1_i1	1.799647938	6.492248671	6.03E-12	3.32E-10	ssaA1
c705_g2_i1	1.305728503	10.59517664	5.44E-10	2.11E-08	tpx
c616_g1_i1	1.302417713	7.583164973	1.48E-08	4.24E-07	truB
c447_g1_i1	-1.767302885	15.3893444	4.48E-17	5.86E-15	vraX
c512_g1_i1	1.09079082	8.836175712	4.16E-07	8.36E-06	xerD
c1764_g1_i1	1.489292565	7.575760991	1.37E-10	6.09E-09	yidC
c104_g1_i1	-2.020830334	4.682259014	1.40E-10	6.11E-09	
c1715_g1_i1	-1.923821164	14.25648051	9.48E-20	1.98E-17	
c842_g1_i1	-1.622474368	3.712816691	2.88E-05	0.000340379	
c493_g3_i1	-1.550062517	7.344481547	8.17E-12	4.27E-10	
c634_g1_i1	-1.473838629	10.24314361	2.67E-12	1.59E-10	
c1145_g1_i1	-1.411793363	6.14612133	1.43E-08	4.15E-07	
c2033_g1_i1	-1.332504673	6.46568065	2.85E-08	7.75E-07	
c419_g2_i1	-1.307792661	7.040379214	1.36E-08	4.00E-07	
c753_g1_i1	-1.272401071	6.930003766	3.95E-08	1.01E-06	
c1425_g1_i1	-1.23169245	5.177484782	1.52E-05	0.000204845	
c299_g2_i1	-1.165112834	5.431667669	1.94E-05	0.000242599	
c2397_g1_i1	-1.149491277	4.932080696	9.88E-05	0.000984543	
c493_g4_i1	-1.130611241	5.16842389	7.73E-05	0.000792584	
c1758_g1_i1	-1.129836386	5.973969951	7.81E-06	0.000111202	
c1087_g1_i1	-1.125226804	11.97449569	5.88E-08	1.40E-06	
c2048_g1_i1	-1.123372801	14.19445501	5.70E-08	1.37E-06	
c599_g1_i1	-1.094359436	10.20839247	1.65E-07	3.56E-06	
c1714_g1_i1	1.001020265	9.412679791	2.38E-06	3.92E-05	
c2364_g1_i1	1.008942784	8.183180639	4.64E-06	6.98E-05	
c2098_g1_i1	1.026877459	6.497150284	4.02E-05	0.000443024	
c245_g1_i1	1.034768963	8.254095988	2.55E-06	4.16E-05	
c721_g1_i1	1.044380595	7.747295664	3.70E-06	5.78E-05	
c765_g1_i1	1.050333845	8.241891078	1.84E-06	3.10E-05	
c712_g1_i1	1.055170272	11.35733562	3.95E-07	8.03E-06	
c72_g2_i1	1.055223002	7.175271648	6.49E-06	9.50E-05	
c431_g1_i1	1.056031754	8.207081751	1.67E-06	2.88E-05	
c632_g2_i1	1.05915718	8.483187131	1.14E-06	2.05E-05	
c2075_g1_i1	1.063831595	7.197055314	5.65E-06	8.33E-05	
c86_g2_i1	1.066828603	11.0182866	3.12E-07	6.40E-06	
c826_g1_i1	1.081709889	6.041099821	4.64E-05	0.000497598	
c1109_g1_i1	1.099570048	6.357635388	1.56E-05	0.000206714	
c2036_g1_i1	1.102185139	6.22329322	2.00E-05	0.000249415	
c736_g1_i1	1.10586719	8.139554771	6.03E-07	1.17E-05	
c380_g1_i1	1.114010508	6.09856768	2.22E-05	0.000273226	
c2127_g1_i1	1.116600523	9.690063148	1.34E-07	2.94E-06	

c1451_g1_i1	1.119331147	8.578265086	2.61E-07	5.41E-06
c2111_g1_i1	1.130080999	8.566796407	2.04E-07	4.31E-06
c516_g1_i1	1.132302816	8.806796444	1.57E-07	3.42E-06
c788_g1_i1	1.146260636	7.112179655	1.26E-06	2.24E-05
c1736_g1_i1	1.151346126	6.726816925	2.65E-06	4.27E-05
c288_g1_i1	1.161061833	5.77795563	2.55E-05	0.000305036
c2452_g1_i1	1.18317839	9.19453546	3.27E-08	8.75E-07
c1712_g1_i1	1.186734995	6.568612674	1.96E-06	3.25E-05
c2520_g1_i1	1.202278666	5.483836419	4.29E-05	0.000464815
c409_g2_i1	1.204518315	9.920728055	1.22E-08	3.66E-07
c810_g1_i1	1.221991986	6.567090682	1.05E-06	1.90E-05
c346_g1_i1	1.259838833	5.631891657	8.98E-06	0.000125191
c657_g2_i1	1.295771117	7.310148559	3.30E-08	8.75E-07
c551_g1_i1	1.297732367	7.782492322	1.14E-08	3.50E-07
c731_g13_i1	1.307853968	9.410729338	9.62E-10	3.66E-08
c3_g1_i1	1.309802279	7.422879848	1.88E-08	5.25E-07
c488_g1_i1	1.309828901	7.837618727	7.48E-09	2.37E-07
c631_g1_i1	1.310641955	6.576000104	1.72E-07	3.67E-06
c697_g2_i1	1.325621082	8.026307911	3.54E-09	1.21E-07
c1157_g1_i1	1.332878963	6.79645486	5.47E-08	1.33E-06
c2032_g1_i1	1.345937474	4.684400214	9.85E-05	0.000984543
c35_g1_i1	1.346478246	8.041256925	1.98E-09	7.28E-08
c0_g1_i1	1.346611216	9.216783613	3.75E-10	1.48E-08
c831_g1_i1	1.361749675	5.103449838	1.74E-05	0.000223804
c789_g1_i1	1.371911815	8.0494127	1.05E-09	3.91E-08
c393_g2_i1	1.380596845	6.830818248	1.96E-08	5.40E-07
c2005_g1_i1	1.3882032	7.463891407	2.45E-09	8.67E-08
c2403_g1_i1	1.396386545	8.837846696	1.33E-10	6.07E-09
c265_g1_i1	1.441678372	8.037612919	1.63E-10	6.82E-09
c156_g2_i1	1.448380707	4.860309774	1.60E-05	0.000209033
c1994_g1_i1	1.45118304	9.01709416	2.13E-11	1.01E-09
c592_g1_i1	1.458737254	10.36670122	5.64E-12	3.19E-10
c1810_g1_i1	1.490416857	7.546240616	1.47E-10	6.27E-09
c1690_g1_i1	1.494655143	8.794793631	7.67E-12	4.11E-10
c2065_g1_i1	1.496477412	7.397124608	2.08E-10	8.55E-09
c2014_g1_i1	1.565510296	6.842277452	2.68E-10	1.08E-08
c1187_g1_i1	1.569692796	7.708600937	1.07E-11	5.45E-10
c2376_g1_i1	1.593473608	5.150778793	6.19E-07	1.19E-05
c2051_g1_i1	1.623282295	6.135242398	2.29E-09	8.28E-08
c737_g1_i1	1.637407729	7.632725222	2.27E-12	1.40E-10
c2427_g1_i1	1.666966865	8.864424831	3.79E-14	3.05E-12
c2394_g1_i1	1.724341599	4.738324636	1.35E-06	2.38E-05
c506_g1_i1	1.788776043	5.298701537	1.83E-08	5.17E-07
c369_g1_i1	1.814946323	8.2593981	1.24E-15	1.37E-13
c2027_g1_i1	1.855036639	11.24975798	2.90E-18	4.05E-16
c2006_g1_i1	1.873834332	11.15951424	1.46E-18	2.54E-16
c1698_g1_i1	1.877564662	10.73539212	1.63E-18	2.62E-16
c274_g1_i1	1.979647335	4.123672915	4.57E-06	6.92E-05
c221_g1_i1	2.00159932	6.21509276	4.54E-13	3.28E-11
c119_g1_i1	2.029653419	6.076087364	6.41E-13	4.47E-11
c759_g1_i1	2.031535783	10.57120093	3.94E-21	1.03E-18
c2015_g1_i1	2.144972268	8.453608589	6.56E-21	1.52E-18
c118_g1_i1	2.154963016	6.655552394	2.75E-16	3.38E-14

c481_g1_i1	2.176572773	7.210928444	2.08E-18	3.10E-16
c2571_g1_i1	2.274773963	8.064917091	3.84E-22	1.15E-19
c884_g1_i1	2.368660248	9.774100831	6.78E-27	3.55E-24
c2367_g1_i1	2.566011187	5.687532606	6.74E-16	7.83E-14
c1114_g1_i1	2.630776058	5.461058947	3.67E-15	3.49E-13
c2423_g1_i2	5.379859727	5.216553563	2.57E-27	1.79E-24
c714_g5_i1	7.966907474	2.24455309	2.85E-05	0.000338449

Supplementary Table S3. Differentially expressed genes in 2R100 compared with 218C06

ID	log2(fold_change)	logCPM	P_Value	FDR	gene name
c549_g1_i1	1.276668955	9.175682492	2.96E-09	3.32E-08	accDA
c794_g1_i1	-1.732929557	9.752038075	4.65E-16	1.23E-14	adh
c608_g1_i1	2.194066108	12.17701804	1.49E-24	1.06E-22	ahpCF
c477_g2_i1	-1.244859117	7.997912283	1.56E-08	1.55E-07	arcDAB
c477_g1_i1	-1.015817283	8.130483583	3.27E-06	2.13E-05	arcR
c73_g1_i1	1.74870823	8.574791911	6.04E-15	1.40E-13	arg
c509_g3_i1	-1.814052524	3.48740982	2.32E-05	0.000121128	argF/arcC1
c2340_g1_i1	-1.868991751	12.50058888	9.87E-19	3.51E-17	argGH
c378_g1_i1	1.126958618	8.224030696	3.82E-07	2.94E-06	argS
c187_g1_i1	1.566262577	8.391420757	2.60E-12	4.23E-11	aroD
c267_g2_i1	-2.786900942	2.787837478	3.71E-07	2.86E-06	arsB
c674_g2_i1	-3.204638812	9.404766412	1.84E-44	7.57E-42	arsRBC
c2424_g1_i1	1.052748184	8.177572606	2.08E-06	1.42E-05	asnS
c443_g2_i1	-3.06609144	2.013454459	2.95E-05	0.000151282	asp2
c111_g1_i2	-1.763530764	3.625812304	1.86E-05	9.94E-05	asp3/secA2
c2371_g2_i1	-1.315631425	12.38671822	2.67E-10	3.34E-09	atl
c2371_g1_i1	-1.225423163	10.67208694	4.66E-09	5.03E-08	atl
c331_g1_i1	1.130866011	9.651108783	9.92E-08	8.62E-07	atl
c147_g1_i1	1.504725851	7.530724552	1.34E-10	1.75E-09	betA
c674_g1_i2	-2.74312151	2.329812766	1.06E-05	6.05E-05	bin3
c2417_g1_i1	-4.526692577	3.106970473	1.99E-14	4.22E-13	bioA
c1757_g1_i1	-1.922736278	3.011863246	9.87E-05	0.000444079	bioB
c434_g1_i1	-3.357258953	11.57023832	1.53E-50	1.05E-47	cadAC
c552_g3_i1	1.538180413	6.356948575	6.46E-09	6.79E-08	cap5A
c29_g1_i1	-1.295382051	5.525220428	2.64E-06	1.76E-05	carA
c343_g1_i1	1.635719654	9.516885912	3.54E-14	7.09E-13	ccpA
c15_g1_i1	-1.471585259	10.35719608	2.93E-12	4.72E-11	clpC
c1699_g1_i1	1.850436223	8.894270725	8.15E-17	2.40E-15	clpX
c589_g1_i1	1.526976478	7.074756578	3.96E-10	4.78E-09	cmk
c731_g1_i1	-2.349297345	9.258579011	1.85E-26	1.53E-24	copA
c350_g1_i1	-1.700075148	9.68776363	1.61E-15	4.01E-14	copB
c731_g5_i1	-2.027736003	7.424503602	2.29E-18	7.75E-17	copZ
c846_g1_i1	1.673088556	5.118314923	3.93E-07	3.01E-06	crcB1
c692_g1_i1	2.110892366	12.0269527	5.93E-23	3.60E-21	cysS/rplK
c768_g1_i1	1.186981845	9.54593429	2.49E-08	2.41E-07	ddl
c466_g1_i1	1.47432425	8.355008908	4.15E-11	5.74E-10	def
c517_g1_i1	-1.077894863	10.43711418	2.46E-07	1.98E-06	dinB/dagK
c1691_g1_i1	1.531996525	11.28089193	3.63E-13	6.92E-12	dltAC
c606_g1_i1	-2.511286529	2.791005022	7.46E-06	4.45E-05	ebh
c358_g2_i1	-2.335594106	2.430587669	0.000128569	0.000563791	ebh
c105_g1_i1	-2.136004806	6.23326383	1.23E-16	3.44E-15	ebhA
c299_g1_i1	-1.872991264	3.706088052	5.38E-06	3.34E-05	ebhA
c123_g1_i1	-1.586521366	4.232362498	6.75E-06	4.08E-05	ebhA
c587_g4_i1	-1.414931892	4.087646189	9.76E-05	0.000440325	ebhA
c2020_g1_i1	4.412301889	9.374303056	3.25E-67	6.70E-64	esxA
c1749_g1_i1	1.402424315	10.41283174	3.34E-11	4.68E-10	fda
c856_g1_i1	1.6096728	8.584110863	4.65E-13	8.63E-12	femBA
c1125_g1_i1	1.502776554	10.24535099	1.54E-12	2.59E-11	fhs
c76_g1_i1	1.416855818	5.471638547	2.31E-06	1.56E-05	fib
c800_g1_i1	-1.628662218	5.593105388	3.19E-09	3.53E-08	flr
c1431_g1_i1	-3.958157564	3.600444595	9.31E-16	2.34E-14	fnbA

c689_g4_i1	-2.874204909	1.863540379	0.000143701	0.00062351	fnbA
c689_g2_i1	-2.295209274	4.119609173	1.16E-09	1.36E-08	fnbA
c177_g1_i1	-2.74312151	2.329812766	1.06E-05	6.05E-05	glcB
c189_g1_i2	10.33290348	4.554095731	1.68E-22	9.87E-21	glcB
c655_g1_i1	1.855278506	7.255651847	3.27E-14	6.61E-13	glcU
c220_g1_i1	2.001501458	8.1967877	4.22E-18	1.34E-16	glpD
c692_g3_i1	1.130910278	8.993716949	1.50E-07	1.26E-06	gltX
c748_g1_i1	1.149080824	12.19873592	3.23E-08	3.10E-07	gluD
c731_g2_i5	1.124361042	9.859505548	1.06E-07	9.16E-07	guaBA
c731_g2_i8	7.311533308	5.61437508	1.93E-36	3.05E-34	guaBA
c619_g1_i1	-2.073916413	7.222906898	1.33E-18	4.63E-17	his
c127_g1_i1	-2.137108666	2.700877051	0.000112452	0.000498416	hla
c167_g1_i1	-1.799645233	4.462850633	1.09E-07	9.36E-07	hlb
c191_g1_i1	-2.680820217	15.12536928	6.66E-35	9.15E-33	hld
c1499_g1_i1	-1.974014169	4.841433428	3.46E-10	4.19E-09	hlgCB
c718_g1_i1	2.969142569	12.93372414	3.33E-41	7.63E-39	hup
c662_g2_i1	2.485601131	10.05143062	2.19E-29	2.38E-27	hutH
c701_g2_i1	-3.044493511	11.21366307	6.69E-43	1.72E-40	hutIU
c501_g1_i1	1.959357536	5.188551227	5.47E-09	5.87E-08	icaR
c501_g2_i1	3.118932966	4.976951645	4.42E-15	1.05E-13	icaR
c1991_g1_i1	2.883944924	10.06290436	3.80E-37	6.52E-35	isaA
c91_g1_i1	3.248701126	2.906333718	3.27E-05	0.000165602	isdC
c2041_g1_i1	1.034862442	7.449210466	7.69E-06	4.55E-05	isdI
c727_g1_i3	1.114672219	10.12446559	1.21E-07	1.04E-06	ispD
c1863_g1_i1	-2.973336074	1.939904286	8.47E-05	0.000387225	kdpA1
c1417_g1_i1	-3.344371049	3.006110239	1.06E-09	1.24E-08	kdpB2
c333_g2_i1	-3.783565331	4.156024692	2.17E-19	8.78E-18	lacAB
c474_g3_i1	-3.541443709	4.724717709	1.82E-22	1.04E-20	lacCBD
c474_g4_i1	-4.188135151	4.653558715	2.37E-27	2.12E-25	lacDFE
c474_g2_i1	-3.064486069	6.154192232	8.76E-29	9.03E-27	lacEG
c243_g1_i1	-1.711749816	10.13125281	7.99E-16	2.03E-14	lip2
c863_g1_i1	-1.78634352	4.654754703	4.07E-08	3.79E-07	lrgAB
c661_g1_i1	1.04380357	8.389136522	1.99E-06	1.38E-05	ltaA/ugtP
c657_g3_i1	1.342011994	7.68987306	5.70E-09	6.05E-08	luxS
c54_g1_i1	2.07277342	9.743224451	2.23E-21	1.15E-19	lytH
c567_g1_i1	1.121496672	7.662163685	9.27E-07	6.65E-06	lytM
c790_g1_i1	1.816766386	7.20206819	1.25E-13	2.45E-12	lytM
c840_g1_i1	-1.955442543	3.163810098	5.09E-05	0.000245569	lytN
c2203_g1_i1	-1.870445382	3.382239224	4.26E-05	0.000209056	lytN
c1817_g1_i1	2.984661784	7.336048614	1.29E-29	1.47E-27	map
c543_g2_i1	-1.153871293	7.469881584	3.01E-07	2.38E-06	metN1
c4_g1_i1	-1.107429632	8.924365634	2.04E-07	1.66E-06	mnhD1
c1144_g1_i1	-1.613267166	5.679741368	2.44E-09	2.78E-08	msa
c1737_g1_i1	1.447073838	7.335538745	1.03E-09	1.21E-08	msrR
c572_g1_i1	-1.406380362	4.180156849	7.38E-05	0.000343563	nanA
c2121_g1_i1	1.066103302	8.215176212	1.49E-06	1.04E-05	nfrA
c429_g1_i1	-1.729638197	5.638395702	2.61E-10	3.28E-09	norB
c665_g2_i1	1.55042833	7.774178324	1.93E-11	2.80E-10	norB
c1394_g1_i1	1.888379878	12.80148798	4.94E-19	1.88E-17	nrdIF
c647_g1_i1	-1.147183508	9.383303555	5.88E-08	5.38E-07	nreCB
c571_g1_i1	1.273233962	7.537571955	4.08E-08	3.79E-07	oatA
c1094_g1_i1	-1.117931036	12.07661126	7.15E-08	6.38E-07	odhBA
c2336_g1_i1	-1.463633726	12.16453071	2.60E-12	4.23E-11	pdxST

c1438_g1_i1	-1.710301139	11.15714319	5.63E-16	1.45E-14	pflAB
c478_g1_i1	-1.306603031	4.859544556	2.24E-05	0.000117613	potA
c479_g1_i1	-1.057088226	9.212664556	6.03E-07	4.50E-06	prmA
c332_g1_i1	2.131128434	7.958887971	1.70E-19	7.33E-18	proC
c1788_g1_i1	-3.536227472	1.841261047	1.48E-05	8.19E-05	pstB
c1566_g1_i1	-7.547349465	1.195708662	9.96E-05	0.000447299	pstS
c513_g2_i1	-1.533976063	4.426942992	7.42E-06	4.45E-05	psuG
c735_g1_i1	-2.425806472	5.79643902	1.45E-18	4.97E-17	ptsG
c1992_g1_i1	2.179399445	7.170543266	4.46E-18	1.37E-16	purA
c2100_g1_i1	1.133818465	8.798266106	1.64E-07	1.37E-06	purB
c394_g1_i1	3.160361506	11.7455048	2.62E-45	1.35E-42	purQLME
c1477_g1_i1	-1.540158814	5.147430976	1.48E-07	1.25E-06	pyrBP
c158_g1_i1	-1.788030469	3.837207887	6.48E-06	3.93E-05	pyrF
c1421_g1_i1	-1.063614029	10.52468291	3.46E-07	2.70E-06	recO
c699_g1_i1	2.557842083	10.58947646	2.91E-31	3.74E-29	ribH
c344_g2_i1	-1.290164451	5.979875908	4.86E-07	3.67E-06	rlmH
c713_g2_i1	1.394176472	8.791942956	1.93E-10	2.44E-09	rnc
c692_g2_i1	1.393310344	10.42477156	4.39E-11	5.99E-10	rplL
c585_g1_i1	1.24294781	13.10174599	2.24E-09	2.58E-08	rplQ
c693_g1_i1	1.612414612	11.89386401	1.92E-14	4.12E-13	rpmB
c117_g2_i1	1.983263105	8.414103812	3.68E-18	1.20E-16	rpmE2
c708_g1_i1	1.255965494	9.917444608	3.13E-09	3.49E-08	rpoZ
c1071_g1_i1	1.218618596	8.887404459	1.80E-08	1.78E-07	rpsB
c225_g1_i1	1.590441792	8.618011107	8.01E-13	1.42E-11	rpsD
c616_g2_i1	1.439831875	9.756522153	1.58E-11	2.33E-10	rpsO
c719_g4_i1	1.03632016	7.963262876	3.70E-06	2.37E-05	rpsP
c46_g1_i1	2.109065286	8.391508965	4.37E-20	1.96E-18	rpsT
c576_g1_i1	1.038800441	10.63307946	6.82E-07	5.00E-06	rpsU
c2501_g1_i1	-1.341059701	6.705675276	2.12E-08	2.07E-07	sarS
c527_g1_i1	-1.162710063	7.225988585	3.64E-07	2.82E-06	sasA
c723_g4_i1	1.776810852	5.64020249	3.83E-09	4.20E-08	sdrC
c723_g2_i1	1.912445186	6.485891392	7.43E-13	1.33E-11	sdrC
c710_g3_i1	1.379949917	5.120169818	1.79E-05	9.65E-05	sdrD
c683_g3_i1	1.011324002	8.141421688	5.15E-06	3.21E-05	spsAB
c720_g1_i1	2.07657875	6.753679782	1.88E-15	4.60E-14	ssaA1
c522_g3_i1	-3.370772003	3.310402302	6.60E-12	1.02E-10	tdcB
c405_g1_i1	1.036400547	9.079212843	1.33E-06	9.36E-06	tig
c68_g1_i1	1.038389645	9.883685214	8.59E-07	6.21E-06	tilS/hpt
c345_g2_i1	-1.709753744	8.6473881	4.99E-15	1.17E-13	tnp
c469_g1_i1	-1.059708058	7.580834219	2.16E-06	1.47E-05	tnpR
c635_g2_i1	1.215873456	8.733232956	2.44E-08	2.37E-07	topB
c1772_g1_i1	-1.189280091	4.722858058	0.000185176	0.000778872	trpBA
c698_g1_i1	1.201508213	9.906200798	1.39E-08	1.39E-07	trxB
c21_g1_i1	1.040665606	7.347799878	7.92E-06	4.65E-05	tsaD
c2442_g1_i1	1.600285472	6.672665592	3.23E-10	3.96E-09	ung
c110_g1_i1	-1.087289381	7.519785006	1.32E-06	9.36E-06	uvrC
c706_g1_i1	1.392201187	9.45157149	8.63E-11	1.15E-09	valS
c1074_g1_i1	-1.334121959	6.212123548	9.19E-08	8.02E-07	vraAC
c447_g1_i1	-1.485452066	15.50354993	1.10E-12	1.90E-11	vraX
c1764_g1_i1	2.598001501	8.506418224	2.14E-28	2.10E-26	yidC
c842_g1_i1	-4.01652723	3.312758385	2.54E-14	5.33E-13	
c647_g2_i1	-3.98932955	6.026248458	3.37E-40	6.94E-38	
c1576_g1_i1	-3.90344785	2.487510507	1.18E-08	1.20E-07	

c2369_g1_i1	-3.847409507	2.940024872	1.42E-11	2.10E-10
c753_g1_i1	-3.842388546	6.546838028	3.57E-43	1.05E-40
c2450_g1_i1	-3.732078193	3.523993513	8.44E-15	1.89E-13
c2048_g1_i1	-3.717771242	13.80013586	6.44E-60	6.64E-57
c643_g1_i1	-3.665766944	3.311573325	5.01E-13	9.14E-12
c956_g1_i1	-3.407243285	3.344868691	4.21E-12	6.71E-11
c574_g1_i1	-3.330647704	1.673963391	8.08E-05	0.000370908
c1773_g1_i1	-3.235426185	2.15282725	6.10E-06	3.76E-05
c178_g2_i1	-3.11419296	3.309245093	3.36E-10	4.09E-09
c2358_g1_i1	-3.082776819	11.32311806	7.85E-44	2.70E-41
c79_g2_i1	-3.040129521	3.007109603	9.20E-09	9.53E-08
c1093_g1_i1	-2.993225452	4.208156713	7.57E-15	1.73E-13
c2410_g1_i1	-2.909306073	2.294081698	1.73E-05	9.41E-05
c844_g1_i1	-2.816898313	2.38902835	6.44E-06	3.92E-05
c124_g2_i1	-2.816898313	2.38902835	6.44E-06	3.92E-05
c1819_g1_i1	-2.805011484	2.532972709	6.16E-06	3.78E-05
c2074_g1_i1	-2.7493678	2.166738602	7.68E-05	0.000354879
c446_g3_i1	-2.680844046	3.683046434	4.18E-10	5.00E-09
c1708_g1_i1	-2.67316239	2.812432696	1.45E-06	1.02E-05
c273_g1_i1	-2.603036535	2.509397226	9.87E-06	5.73E-05
c2131_g1_i1	-2.583183237	2.205760213	4.68E-05	0.000227832
c420_g1_i1	-2.543078621	3.497216077	1.29E-08	1.29E-07
c157_g2_i1	-2.516363854	2.304870061	0.000103608	0.000463201
c1425_g1_i1	-2.424150562	4.917398115	2.74E-14	5.59E-13
c95_g2_i1	-2.415502237	3.088875724	7.85E-07	5.73E-06
c1397_g1_i1	-2.401260691	8.983662915	3.67E-27	3.15E-25
c446_g2_i1	-2.365819189	2.568896545	3.36E-05	0.000169499
c1436_g1_i1	-2.350737681	3.338297996	2.81E-07	2.23E-06
c452_g1_i1	-2.332519605	3.253640974	8.98E-07	6.47E-06
c420_g5_i1	-2.317216186	3.728484548	2.95E-08	2.84E-07
c59_g1_i1	-2.288600492	9.998348194	4.44E-26	3.39E-24
c2373_g1_i1	-2.230092433	3.493240174	3.15E-07	2.47E-06
c217_g1_i1	-2.202070731	4.804784943	6.36E-12	9.94E-11
c217_g2_i1	-2.150807267	5.538951994	2.60E-14	5.42E-13
c1479_g1_i1	-2.148526313	4.255179681	3.86E-09	4.21E-08
c894_g1_i1	-2.125231194	5.689511005	1.42E-14	3.12E-13
c2145_g1_i1	-2.114608878	2.772870918	4.94E-05	0.000239582
c125_g1_i1	-2.054180987	6.068945049	3.76E-15	9.02E-14
c82_g1_i1	-2.045930916	8.711454523	1.74E-20	8.54E-19
c398_g2_i1	-2.036215241	3.988652258	7.95E-08	7.04E-07
c793_g1_i1	-2.033036935	2.79714513	0.000124562	0.00054855
c868_g1_i1	-1.991830677	8.657705381	1.71E-19	7.33E-18
c599_g1_i1	-1.978890237	10.02444203	2.72E-20	1.27E-18
c196_g1_i1	-1.969922181	3.602869801	3.29E-06	2.13E-05
c161_g1_i1	-1.969922181	3.602869801	3.29E-06	2.13E-05
c1697_g1_i1	-1.964216898	7.057365016	1.05E-16	3.01E-15
c299_g2_i1	-1.959259185	5.252971798	2.64E-11	3.75E-10
c664_g2_i1	-1.952387261	9.106771331	3.26E-19	1.29E-17
c1162_g1_i1	-1.932504941	3.574688018	6.32E-06	3.87E-05
c568_g2_i1	-1.904822277	10.50387521	4.05E-19	1.57E-17
c462_g2_i1	-1.897004271	3.181699611	7.67E-05	0.000354879
c874_g1_i1	-1.856692607	4.123807111	4.69E-07	3.55E-06
c419_g2_i1	-1.845402723	6.945826309	9.10E-15	2.02E-13

c453_g1_i1	-1.823487252	3.826558175	4.55E-06	2.90E-05
c529_g3_i2	-1.808360195	4.62891657	3.72E-08	3.54E-07
c1112_g1_i1	-1.800948004	3.652970892	1.37E-05	7.65E-05
c1247_g1_i1	-1.795389287	3.729398233	7.82E-06	4.60E-05
c547_g2_i1	-1.782397263	5.430328573	3.07E-10	3.81E-09
c493_g3_i1	-1.772756251	7.336099329	1.83E-14	3.97E-13
c87_g1_i1	-1.763530764	3.625812304	1.86E-05	9.94E-05
c2411_g1_i1	-1.748465773	5.673360032	1.37E-10	1.77E-09
c645_g2_i1	-1.736340497	5.743725016	1.09E-10	1.45E-09
c270_g2_i1	-1.729311065	3.426824487	6.11E-05	0.000290081
c252_g1_i1	-1.727775525	4.631467817	1.71E-07	1.42E-06
c2116_g1_i1	-1.721749282	3.752228343	1.49E-05	8.25E-05
c652_g2_i1	-1.706692023	13.18760551	4.78E-16	1.25E-14
c422_g1_i1	-1.701589978	7.080996718	3.71E-13	7.01E-12
c2118_g1_i1	-1.688350262	4.642821352	2.27E-07	1.82E-06
c587_g3_i1	-1.687196592	4.088470396	4.64E-06	2.95E-05
c529_g1_i1	-1.685176199	3.441295391	0.00011673	0.000516266
c2505_g1_i1	-1.662579152	4.261811956	3.22E-06	2.11E-05
c607_g1_i1	-1.65418389	8.78191262	2.72E-14	5.59E-13
c1709_g1_i1	-1.65348491	3.844871384	2.12E-05	0.000112131
c1755_g1_i1	-1.600761486	3.469683054	0.000214124	0.000880859
c215_g1_i1	-1.600182639	4.765948113	3.62E-07	2.81E-06
c726_g2_i1	-1.577312674	7.483469872	4.42E-12	6.95E-11
c293_g1_i1	-1.562351362	4.240073544	1.12E-05	6.38E-05
c480_g2_i1	-1.542225767	3.698333792	0.000197693	0.000822119
c2493_g1_i1	-1.517370733	9.428229648	1.07E-12	1.86E-11
c1778_g1_i1	-1.493257642	8.340903798	8.86E-12	1.35E-10
c2051_g1_i1	-1.479076837	4.491640325	7.57E-06	4.49E-05
c458_g1_i1	-1.464234318	7.07438133	3.13E-10	3.86E-09
c494_g1_i1	-1.463181543	15.03281482	2.31E-12	3.80E-11
c141_g1_i1	-1.45995669	8.76906113	1.39E-11	2.08E-10
c1822_g1_i1	-1.459857296	3.839147206	0.000182622	0.000769701
c452_g5_i1	-1.384039243	4.612559678	2.11E-05	0.000112131
c1462_g1_i1	-1.365724687	6.328302484	3.98E-08	3.75E-07
c1715_g1_i1	-1.354857869	14.44033956	7.42E-11	9.92E-10
c1704_g1_i1	-1.34828151	7.429285904	2.71E-09	3.06E-08
c150_g2_i1	-1.32571972	4.149517666	0.000224545	0.000918228
c572_g4_i1	-1.314169152	4.296742589	0.000192717	0.00080566
c801_g1_i1	-1.267584849	6.142581352	4.58E-07	3.48E-06
c520_g1_i1	-1.212163044	5.336889409	1.58E-05	8.69E-05
c302_g1_i1	-1.209231647	7.090046367	1.72E-07	1.42E-06
c395_g1_i1	-1.2010939	11.11232069	8.45E-09	8.80E-08
c349_g3_i1	-1.194017988	5.473833022	1.41E-05	7.88E-05
c574_g2_i1	-1.193109534	7.741583496	8.38E-08	7.35E-07
c1472_g1_i1	-1.186421561	8.062612385	6.50E-08	5.93E-07
c579_g1_i1	-1.175702144	6.008390592	4.23E-06	2.71E-05
c32_g1_i1	-1.142237225	9.244126795	7.14E-08	6.38E-07
c1091_g1_i1	-1.1407286	15.09762997	3.56E-08	3.39E-07
c1100_g1_i1	-1.138388386	9.570280609	6.81E-08	6.15E-07
c2244_g1_i1	-1.069593152	6.901643292	5.05E-06	3.17E-05
c69_g1_i1	-1.06458463	12.57666409	2.73E-07	2.17E-06
c116_g2_i1	-1.053557709	5.932919897	4.26E-05	0.000209056
c2061_g1_i1	-1.051824276	9.751427991	5.59E-07	4.19E-06

c300_g1_i1	-1.029721616	10.42072934	8.08E-07	5.86E-06
c370_g1_i1	-1.026239607	6.986495773	1.03E-05	5.94E-05
c731_g10_i1	-1.009435767	6.198059822	5.17E-05	0.000249079
c1763_g1_i1	1.006821286	6.339255501	8.95E-05	0.000407174
c2446_g1_i1	1.016502543	8.063917414	5.11E-06	3.20E-05
c1401_g1_i1	1.021482676	10.11108284	1.18E-06	8.45E-06
c144_g1_i1	1.024352403	7.455608669	8.86E-06	5.17E-05
c1066_g1_i1	1.0256385	7.609110035	7.48E-06	4.45E-05
c2354_g1_i1	1.035169737	8.804428248	1.64E-06	1.14E-05
c181_g1_i1	1.04709513	7.106539022	1.04E-05	5.99E-05
c505_g2_i1	1.047747937	5.652316291	0.000236857	0.000962843
c369_g1_i1	1.065042357	7.757200721	2.64E-06	1.76E-05
c1765_g1_i1	1.072101991	7.640534887	2.69E-06	1.79E-05
c1101_g1_i1	1.086806901	8.382409233	7.86E-07	5.73E-06
c2014_g1_i1	1.093154445	6.548553975	1.41E-05	7.88E-05
c427_g2_i1	1.102583735	6.082085753	3.68E-05	0.000183789
c559_g2_i1	1.103464298	7.261037056	2.71E-06	1.80E-05
c551_g1_i1	1.105995388	7.693513241	1.19E-06	8.49E-06
c322_g1_i1	1.122844711	5.655624132	9.15E-05	0.000415396
c530_g1_i1	1.12405116	7.83039029	6.72E-07	4.95E-06
c2005_g1_i1	1.130600245	7.326702702	1.38E-06	9.69E-06
c2359_g1_i1	1.132333262	9.08184685	1.36E-07	1.15E-06
c2033_g1_i1	1.13539327	7.737146961	6.22E-07	4.63E-06
c2095_g1_i1	1.137237657	8.666497813	1.79E-07	1.46E-06
c72_g2_i1	1.148238504	7.283846015	1.00E-06	7.17E-06
c675_g3_i1	1.150090006	9.499831602	6.62E-08	6.01E-07
c1522_g1_i1	1.154062976	8.299424176	1.82E-07	1.48E-06
c1812_g1_i1	1.168294051	9.61077448	3.89E-08	3.68E-07
c595_g2_i1	1.168306573	5.261697985	0.000154634	0.000663961
c2036_g1_i1	1.172668774	6.31665998	6.77E-06	4.08E-05
c35_g1_i1	1.176883555	7.966372203	1.72E-07	1.42E-06
c737_g1_i1	1.205691357	7.363286661	2.65E-07	2.12E-06
c657_g4_i1	1.206901325	6.519370231	2.03E-06	1.40E-05
c565_g1_i1	1.209407672	9.767282552	1.21E-08	1.22E-07
c2426_g1_i1	1.226170587	5.822691553	1.23E-05	7.00E-05
c1092_g1_i1	1.23059556	6.792041885	6.39E-07	4.73E-06
c1981_g1_i1	1.241071717	6.689788808	6.44E-07	4.75E-06
c118_g1_i1	1.241506866	6.006381256	5.48E-06	3.39E-05
c2075_g1_i1	1.25782573	7.376333366	8.34E-08	7.34E-07
c521_g2_i1	1.268184366	6.72153661	3.34E-07	2.61E-06
c570_g1_i1	1.278048412	8.153921337	1.12E-08	1.14E-07
c593_g1_i1	1.287634827	8.447540774	5.67E-09	6.05E-08
c1121_g1_i1	1.289147595	8.763503938	3.50E-09	3.86E-08
c955_g1_i1	1.29366909	9.017297836	2.15E-09	2.49E-08
c2032_g1_i1	1.307131869	4.699831688	0.000211398	0.000871384
c1690_g1_i1	1.307751827	8.704219453	2.34E-09	2.68E-08
c1408_g1_i1	1.315276747	6.690889093	1.60E-07	1.34E-06
c1736_g1_i1	1.320260973	6.890358036	7.53E-08	6.69E-07
c2348_g1_i1	1.341321407	7.527824233	8.33E-09	8.71E-08
c538_g1_i1	1.341736632	6.905735589	4.59E-08	4.22E-07
c71_g1_i1	1.348483059	6.577147187	1.24E-07	1.06E-06
c85_g1_i1	1.363740446	7.042274811	1.82E-08	1.79E-07
c590_g1_i1	1.373538729	5.032103214	3.00E-05	0.000153635

c0_g1_i1	1.376232763	9.283152385	1.61E-10	2.05E-09
c631_g1_i1	1.381962722	6.671675621	4.04E-08	3.79E-07
c424_g1_i2	1.382496864	4.855437088	6.62E-05	0.000312299
c218_g1_i1	1.383613151	5.239460157	1.24E-05	7.03E-05
c337_g1_i1	1.384346344	7.078045885	1.03E-08	1.06E-07
c788_g1_i1	1.388393999	7.328255803	4.53E-09	4.91E-08
c168_g1_i1	1.3945169	4.762884588	7.37E-05	0.000343563
c1157_g1_i1	1.401086291	6.890134857	1.29E-08	1.29E-07
c380_g1_i1	1.415660406	6.356723687	6.94E-08	6.24E-07
c2364_g1_i1	1.416555488	8.513146652	1.56E-10	2.00E-09
c809_g1_i1	1.419978877	9.978120638	2.49E-11	3.56E-10
c1752_g1_i1	1.421892318	6.963654417	5.94E-09	6.28E-08
c1079_g1_i1	1.422086313	5.507354083	2.04E-06	1.40E-05
c1507_g1_i1	1.425153138	11.67249426	1.07E-11	1.61E-10
c316_g1_i1	1.431828858	6.41976098	4.26E-08	3.93E-07
c516_g1_i1	1.433337956	9.065229227	3.77E-11	5.25E-10
c756_g1_i1	1.443894278	4.44263524	0.000154621	0.000663961
c1698_g1_i1	1.467335266	10.46848203	4.23E-12	6.71E-11
c366_g2_i1	1.47098994	9.225856327	1.00E-11	1.52E-10
c3_g2_i1	1.475800515	4.247252351	0.000238022	0.000965678
c576_g2_i1	1.483376715	8.506415284	2.29E-11	3.30E-10
c524_g2_i1	1.516650157	6.888792698	9.25E-10	1.10E-08
c1126_g1_i1	1.521723316	10.65646556	6.57E-13	1.19E-11
c406_g1_i1	1.523537438	4.501196787	6.87E-05	0.000321803
c1997_g1_i1	1.533955332	4.439696773	7.23E-05	0.0003379
c772_g1_i1	1.554989451	7.576030817	3.12E-11	4.41E-10
c2403_g1_i1	1.560836777	9.003793612	1.01E-12	1.77E-11
c26_g2_i1	1.568610645	7.013351068	1.61E-10	2.05E-09
c430_g1_i1	1.594695702	8.141518674	2.07E-12	3.44E-11
c559_g1_i1	1.596860345	7.730127549	6.70E-12	1.03E-10
c765_g1_i1	1.60804348	8.685655717	4.05E-13	7.59E-12
c1451_g1_i1	1.62144858	8.985151448	1.49E-13	2.90E-12
c2468_g1_i1	1.634448322	6.938252377	5.10E-11	6.87E-10
c221_g1_i1	1.668770032	6.00113064	2.58E-09	2.92E-08
c2376_g1_i1	1.682291017	5.260368887	1.77E-07	1.45E-06
c614_g1_i1	1.693035206	7.407376389	1.39E-12	2.39E-11
c2013_g1_i1	1.704951439	7.326576783	1.49E-12	2.53E-11
c433_g1_i1	1.7152982	13.39668069	3.63E-16	9.84E-15
c450_g2_i1	1.718138754	4.949544256	5.32E-07	4.00E-06
c2477_g1_i1	1.718878044	9.257243762	2.92E-15	7.09E-14
c2394_g1_i1	1.729591374	4.784433939	1.73E-06	1.20E-05
c1238_g1_i1	1.737498443	4.592199347	5.19E-06	3.23E-05
c757_g1_i1	1.766844864	5.486868955	1.03E-08	1.06E-07
c511_g1_i1	1.769683994	7.698084507	5.10E-14	1.01E-12
c224_g1_i1	1.786631304	5.871672962	6.09E-10	7.25E-09
c384_g1_i1	1.786929426	5.001885394	2.24E-07	1.81E-06
c1187_g1_i1	1.799993124	7.928464091	8.37E-15	1.89E-13
c1109_g1_i1	1.821745803	6.930746	4.83E-13	8.90E-12
c418_g1_i1	1.851369468	3.825657497	8.45E-05	0.000387145
c731_g13_i1	1.913890122	9.911673871	9.29E-19	3.36E-17
c250_g1_i1	1.927424846	11.2213548	1.75E-19	7.38E-18
c778_g1_i1	1.936769549	7.634539634	4.43E-16	1.19E-14
c2007_g1_i1	1.971203664	9.015526901	8.46E-19	3.17E-17

c2085_g1_i1	1.975352031	7.683802343	1.05E-16	3.01E-15
c2065_g1_i1	2.026971343	7.849701956	9.52E-18	2.88E-16
c2136_g1_i1	2.027266431	4.498048406	3.96E-07	3.02E-06
c3_g1_i1	2.028047544	8.011986959	4.04E-18	1.30E-16
c2427_g1_i1	2.042848134	9.203213404	3.60E-20	1.65E-18
c810_g1_i1	2.045784015	7.231509611	1.78E-16	4.89E-15
c1393_g1_i1	2.051090968	5.456653261	1.25E-10	1.63E-09
c2520_g1_i1	2.05195252	6.16515624	3.02E-13	5.82E-12
c2464_g1_i1	2.066404259	9.789039704	2.68E-21	1.35E-19
c278_g1_i1	2.15593496	7.752213906	2.02E-19	8.33E-18
c481_g1_i1	2.172798331	7.251963889	3.10E-18	1.03E-16
c2043_g1_i1	2.18043684	7.171385349	4.46E-18	1.37E-16
c733_g1_i1	2.189102612	11.38541391	2.75E-24	1.89E-22
c2367_g1_i1	2.195586737	5.427017001	1.63E-11	2.39E-10
c789_g1_i1	2.216422116	8.747182702	2.09E-22	1.16E-20
c836_g1_i1	2.222458148	9.760028947	4.39E-24	2.92E-22
c722_g1_i1	2.245201412	14.96985705	9.25E-26	6.81E-24
c721_g1_i1	2.266336551	8.715004265	3.59E-23	2.31E-21
c346_g1_i1	2.284262154	6.459988998	1.18E-16	3.34E-15
c200_g2_i1	2.286445784	4.290609274	1.37E-07	1.16E-06
c731_g4_i2	2.304878148	7.373953932	2.45E-20	1.18E-18
c393_g2_i1	2.331811154	7.61514263	1.56E-21	8.27E-20
c657_g2_i1	2.334372937	8.158737192	4.22E-23	2.64E-21
c2006_g1_i1	2.387131948	11.62203122	3.09E-28	2.90E-26
c104_g1_i1	2.459935753	7.227607154	7.35E-22	3.98E-20
c1078_g1_i1	2.592616312	6.224778602	8.86E-19	3.26E-17
c1983_g1_i1	2.609099382	7.811726709	2.59E-26	2.05E-24
c400_g2_i1	2.61328422	3.248291672	5.42E-05	0.000259562
c156_g2_i1	2.628462105	5.831779843	7.16E-17	2.14E-15
c274_g1_i1	2.672338769	4.718817381	4.21E-11	5.78E-10
c2066_g1_i1	2.885876101	4.514476971	5.03E-11	6.82E-10
c2571_g1_i1	3.150241877	8.863509588	1.37E-39	2.57E-37
c2153_g1_i1	3.225687801	2.887611605	3.27E-05	0.000165602
c2399_g1_i1	3.514214172	6.54996591	1.58E-30	1.92E-28
c89_g1_i1	4.113520839	2.792222947	8.72E-06	5.11E-05
c2423_g1_i2	5.903448345	5.741855212	2.19E-35	3.22E-33
c715_g1_i1	7.854946698	2.400064335	1.03E-05	5.95E-05
c714_g5_i1	9.186164614	3.535633747	1.99E-12	3.34E-11

Supplementary Table S4. Differentially expressed genes in 2R150 compared with 236C05

ID	log2(fold_change)	logCPM	P_Value	FDR	gene name
c637_g2_i1	-1.726047341	11.6734329	9.19E-16	1.60E-14	acnA
c2383_g1_i1	1.373004509	7.936939594	3.06E-05	0.000173261	acuC
c139_g1_i1	-1.251576072	10.02914544	2.79E-08	2.28E-07	acyP
c794_g1_i1	14.7787134	9.327961673	1.17E-57	1.34E-55	adh
c556_g1_i1	6.996937703	13.52439333	7.06E-129	7.30E-126	agrA
c608_g1_i1	-2.271378269	12.68709673	7.02E-26	2.38E-24	ahpCF
c526_g1_i1	3.482613037	8.714174674	1.25E-23	3.80E-22	araB
c477_g2_i1	4.730618444	6.386781152	2.72E-09	2.55E-08	arcAB
c2340_g1_i1	2.472046645	9.41956253	6.44E-19	1.33E-17	argGH
c187_g1_i1	-1.499419829	6.664516452	5.97E-05	0.00032877	aroD
c674_g2_i1	10.77392838	5.766629585	1.68E-09	1.63E-08	arsRBC
c1381_g1_i1	1.663422167	10.63477439	7.60E-13	1.03E-11	asp23
c2371_g1_i1	3.497240079	7.368159118	7.19E-13	9.79E-12	atl
c1780_g1_i1	4.859616987	6.496162663	4.98E-10	5.18E-09	bbp
c1705_g1_i1	-12.81730144	6.826219082	6.09E-31	2.74E-29	blaZ
c555_g1_i1	1.509745969	7.195277171	0.000171129	0.000874236	bsaA
c434_g1_i1	1.952980256	9.686416159	3.80E-14	5.87E-13	cadAC
c1520_g1_i1	-10.73048808	3.110287683	2.21E-08	1.82E-07	cadC
c552_g4_i1	2.202069217	8.050997199	2.49E-10	2.62E-09	cap5A
c343_g1_i1	-1.056218916	8.510310653	5.23E-05	0.000289984	ccpA
c1386_g1_i1	-1.758718557	8.915688925	1.28E-12	1.66E-11	cdr
c40_g1_i1	1.870136421	7.558625022	7.05E-07	4.91E-06	cidB
c710_g2_i2	-11.53747904	4.746340212	8.98E-14	1.33E-12	clfA
c710_g2_i1	9.908678541	5.026986544	7.29E-06	4.48E-05	clfA
c743_g1_i1	1.209042489	14.32212091	7.05E-09	6.29E-08	clpB
c15_g2_i1	-1.011032312	13.35950154	1.18E-06	8.09E-06	clpC
c1083_g1_i1	12.4220129	7.183064176	1.27E-21	3.25E-20	clpL
c1699_g1_i1	-3.123283655	9.171139448	1.19E-35	6.15E-34	clpX
c630_g1_i1	1.761126151	6.85122511	0.000132083	0.000679799	cls2
c589_g1_i1	4.158502966	5.903864991	3.33E-06	2.13E-05	cmk
c690_g1_i1	-2.355867025	7.243264943	6.20E-13	8.55E-12	cna
c185_g1_i1	-1.884094388	6.820809971	1.37E-07	1.03E-06	coaD
c731_g1_i1	-1.677426668	8.168663984	8.18E-10	8.29E-09	copA
c350_g1_i1	5.479978514	7.026478478	4.95E-15	8.00E-14	copB
c731_g5_i1	-2.121842008	5.368683918	3.67E-05	0.000204888	copZ
c342_g2_i1	-1.567129356	7.87161923	4.65E-08	3.72E-07	cysK
c733_g2_i1	1.680157629	7.233533492	3.66E-05	0.000204671	dapABH
c90_g1_i1	10.26605323	5.333167799	2.72E-07	1.99E-06	dapE
c768_g1_i1	1.654273979	8.5296597	2.81E-08	2.29E-07	ddl
c715_g2_i1	1.156864486	8.465322716	6.49E-05	0.000355088	deoC1
c517_g1_i1	2.330038264	9.757883007	8.23E-19	1.69E-17	dinB/dagK
c562_g1_i1	-1.220434258	8.462191628	2.99E-06	1.93E-05	dinG/cca
c105_g1_i1	10.23155392	5.303664732	5.52E-07	3.87E-06	ebhA
c1110_g1_i1	-1.190863775	7.659402003	7.39E-05	0.000400111	efp
c1831_g1_i1	-10.19030562	2.216959066	2.48E-06	1.63E-05	ermA
c2020_g1_i1	1.568512589	7.62614697	1.28E-05	7.57E-05	esxA
c713_g1_i1	-1.245052078	8.944645207	4.30E-07	3.07E-06	fabDG
c628_g1_i1	-1.403420689	7.347599368	1.07E-05	6.41E-05	fabHF
c524_g1_i1	3.998679754	7.140062707	6.03E-13	8.38E-12	fbp
c1749_g1_i1	-1.110717214	9.879915273	1.01E-06	6.92E-06	fda
c577_g3_i1	-1.747004623	7.246489394	6.59E-08	5.24E-07	fdhD

c1996_g1_i1	-1.265845869	9.140602176	1.57E-07	1.17E-06	femA
c856_g1_i1	4.272099407	6.860328148	1.94E-11	2.27E-10	femBA
c1125_g1_i1	-1.675310589	9.780185274	3.12E-13	4.55E-12	fhs
c1139_g1_i1	10.7909519	5.781142757	1.22E-09	1.21E-08	fmtA
c1420_g1_i1	2.099161528	8.393065953	4.90E-11	5.51E-10	fumC
c598_g1_i1	-2.997783287	6.700850408	2.27E-15	3.75E-14	fur
c228_g1_i1	-2.621762565	12.1526391	6.43E-33	3.10E-31	fusA/rpsL
c2010_g1_i1	5.258317199	10.76644679	1.07E-65	1.58E-63	gapA2
c627_g1_i1	-1.714565645	8.230158966	2.28E-10	2.45E-09	gatAB
c189_g1_i2	-4.654737024	3.87460747	2.46E-10	2.60E-09	glcB
c655_g1_i1	-1.907995602	7.105047016	1.28E-08	1.10E-07	glcU
c136_g2_i1	1.885117682	8.275853338	4.06E-09	3.70E-08	glmS
c136_g1_i1	4.423013417	7.503812096	4.72E-17	9.12E-16	glmS
c220_g1_i1	1.360508446	9.503480287	7.45E-08	5.79E-07	glpD
c1118_g1_i1	-1.97417795	8.895415106	2.04E-15	3.42E-14	glyQS
c238_g1_i1	-1.648478413	14.94938293	4.26E-15	7.00E-14	gpsB
c557_g1_i1	1.993426612	7.158710053	3.82E-06	2.42E-05	graRS
c670_g2_i1	-2.537196105	9.428905738	4.59E-26	1.61E-24	greA
c594_g1_i1	4.252763919	11.5344138	3.06E-59	3.72E-57	groLS
c1701_g1_i1	1.557281914	10.17179238	7.90E-11	8.74E-10	hemA
c2402_g1_i1	-1.386285537	6.714939475	0.000193665	0.00097969	hemL2
c191_g1_i1	6.172557574	11.85759155	3.90E-93	1.35E-90	hld
c68_g1_i1	-2.619192857	9.628208695	3.01E-28	1.24E-26	hpt
c342_g1_i1	-2.432115666	6.942508716	3.01E-12	3.83E-11	hslO
c561_g1_i1	2.135682307	6.482560363	9.85E-05	0.000521061	hutG
c701_g2_i1	5.99022704	9.599722797	2.11E-52	1.82E-50	hutIU
c324_g1_i1	-1.606726301	11.7119305	6.09E-14	9.13E-13	icd
c660_g1_i1	1.865574955	6.794782812	9.05E-05	0.000480297	ilvDC
c545_g1_i1	2.38891988	7.474542721	4.95E-09	4.48E-08	ilvE
c1991_g1_i1	-3.314407937	9.507852956	2.63E-41	1.60E-39	isaA
c1753_g1_i1	1.162282675	8.275144684	0.000115088	0.000596783	isaB
c727_g1_i3	-1.296963077	9.146691793	7.10E-08	5.58E-07	ispD
c2342_g1_i1	-2.204935653	11.79727077	3.20E-24	1.02E-22	katA
c333_g2_i1	9.524992893	4.696603688	8.39E-05	0.000448565	lacAB
c474_g4_i1	9.549207801	4.717511331	8.39E-05	0.000448565	lacDFE
c474_g2_i1	10.81111848	5.798335191	2.17E-09	2.07E-08	lacEG
c2112_g1_i1	4.313300571	9.126343549	2.23E-34	1.10E-32	ldhA
c1724_g1_i1	2.783472068	10.46723526	5.55E-28	2.21E-26	lexA
c243_g1_i1	3.637532115	6.834918969	7.38E-10	7.52E-09	lip2
c654_g1_i1	-1.706898066	9.430136249	4.34E-13	6.11E-12	lipA
c145_g1_i1	-1.3390606	10.13836707	2.36E-09	2.23E-08	ltaS
c54_g1_i1	-2.232945538	9.295104181	1.66E-20	3.73E-19	lytH
c567_g1_i1	-2.517173906	7.324804469	6.98E-15	1.12E-13	lytM
c1817_g1_i1	2.722828648	6.075658004	7.05E-05	0.000382716	map
c543_g2_i1	10.22154353	5.295102132	5.52E-07	3.87E-06	metN1
c1729_g1_i1	3.084375732	8.983063143	1.72E-22	4.62E-21	mgt
c553_g1_i1	-1.407565569	9.515099629	1.70E-09	1.64E-08	mnhC1
c4_g1_i1	4.426539681	6.991744325	8.81E-13	1.18E-11	mnhD1
c695_g1_i1	-1.88303324	6.92507767	8.09E-08	6.27E-07	mntH
c687_g4_i1	2.463560355	7.649746635	4.06E-10	4.25E-09	moaCB
c687_g2_i1	-1.315401608	8.61570458	2.81E-07	2.04E-06	mobA
c1447_g1_i1	1.92362796	7.218488527	4.23E-06	2.65E-05	mprF
c1805_g1_i1	1.328458008	8.862318353	1.38E-06	9.29E-06	mqo1

c170_g1_i1	2.364210248	8.379668446	6.03E-13	8.38E-12	murQ
c677_g1_i1	-2.329368707	10.83118352	1.88E-25	6.27E-24	mutS2/trxA
c694_g1_i1	-1.323473333	7.994224409	2.46E-06	1.63E-05	nadK
c656_g1_i1	2.17417192	7.956075967	8.33E-10	8.40E-09	norA
c1394_g1_i1	2.590366341	10.52269314	2.38E-25	7.82E-24	nrdIF
c647_g1_i1	5.951816889	7.436175217	7.99E-20	1.76E-18	nreCB
c817_g1_i1	-14.37356286	8.690694402	1.21E-78	3.14E-76	pbp
c1253_g1_i1	-12.72985109	6.707211018	2.73E-29	1.18E-27	pbp
c921_g1_i1	-12.22179385	5.960883509	3.17E-21	7.70E-20	pbp
c1469_g1_i1	-10.90031873	3.439471201	1.30E-09	1.27E-08	pbp
c442_g1_i1	-3.612726274	12.37759657	9.00E-56	9.21E-54	pdhBCD
c2336_g1_i1	6.155187765	10.97987766	2.65E-78	6.10E-76	pdxST
c1438_g1_i1	7.244731236	12.15795346	2.61E-110	1.08E-107	pflAB
c726_g1_i1	8.926535997	10.18351985	5.55E-74	9.56E-72	pre
c685_g1_i1	-1.523291276	8.714565556	1.78E-09	1.71E-08	prkC/fmt
c2430_g1_i1	3.23679624	8.681401476	1.91E-21	4.77E-20	prsA
c394_g1_i1	2.725987224	9.568183655	1.35E-22	3.68E-21	purQLMEDC
c671_g1_i1	-2.524690665	8.669531173	1.27E-22	3.52E-21	queA
c1402_g1_i1	-1.734732048	6.474639125	1.14E-05	6.77E-05	rbfA
c26_g1_i1	3.656060027	10.91326771	2.33E-44	1.55E-42	recA
c2038_g1_i1	-1.655925042	9.898477725	3.93E-13	5.61E-12	recU
c642_g1_i1	-3.863404308	9.397333664	8.20E-52	6.79E-50	ribU
c713_g2_i1	-1.94492385	8.164377104	1.11E-12	1.47E-11	rnc
c1703_g1_i1	-1.409029577	10.95797677	7.96E-11	8.76E-10	rny
c1207_g1_i1	-1.627948749	7.394862247	2.79E-07	2.03E-06	rot
c692_g2_i1	-1.987596492	9.309875965	9.30E-17	1.78E-15	rplL
c618_g1_i1	-1.426604585	8.959102266	6.57E-09	5.89E-08	rplM
c719_g3_i1	-3.271950913	9.19128062	1.14E-38	6.38E-37	rplS
c716_g3_i1	-2.123421006	7.582537345	2.51E-12	3.23E-11	rplT
c2059_g1_i1	-1.910977105	9.60921968	2.87E-16	5.21E-15	rpmA
c693_g1_i1	-2.22622254	10.65813419	2.53E-23	7.47E-22	rpmB
c117_g2_i1	-2.717042001	8.341798387	9.44E-24	2.91E-22	rpmE2
c1688_g1_i1	-1.750073211	8.679440244	5.30E-12	6.64E-11	rpmF
c1985_g1_i1	-1.183204627	11.73056168	2.51E-08	2.06E-07	rpoBC
c622_g1_i1	-2.038447541	6.969202726	3.94E-09	3.62E-08	rpoE
c1071_g1_i1	-2.365328378	8.338964381	1.09E-18	2.22E-17	rpsB
c225_g1_i1	-2.783609205	7.884043626	1.35E-21	3.42E-20	rpsD
c421_g1_i1	-2.293405275	9.580291125	2.76E-22	7.33E-21	rpsF
c585_g1_i1	-1.926844882	12.99479366	1.58E-19	3.36E-18	rpsJ
c1183_g1_i1	-3.404643301	4.692325448	1.09E-08	9.47E-08	rpsN
c616_g2_i1	-3.463156781	9.718022208	1.92E-45	1.32E-43	rpsO
c46_g1_i1	-2.216469034	8.335402381	1.45E-16	2.70E-15	rpsT
c576_g1_i1	-2.890486954	10.09740681	5.28E-35	2.66E-33	rpsU
c1378_g1_i1	1.96620943	9.574880985	5.48E-14	8.27E-13	rsmH
c671_g2_i1	-1.292249026	7.87479441	6.44E-06	3.98E-05	ruvBA
c451_g1_i1	-1.60909329	7.349891959	4.49E-07	3.19E-06	saeSR
c367_g2_i1	3.289433479	6.544043711	1.09E-07	8.33E-07	sarV
c731_g6_i1	3.336803679	7.233076001	1.84E-11	2.18E-10	sarZ
c2107_g1_i1	2.682958562	7.124332831	1.09E-08	9.47E-08	scdA
c723_g2_i1	10.26605323	5.333167799	2.72E-07	1.99E-06	sdrC
c723_g1_i1	10.44448074	5.485608116	1.02E-07	7.84E-07	sdrD
c723_g5_i1	10.92035853	5.891464743	6.02E-10	6.20E-09	sdrD
c1719_g1_i1	1.531329202	10.10672148	1.96E-10	2.12E-09	secA2

c1411_g1_i1	4.39833862	10.59728676	1.11E-52	1.00E-50	sepA
c534_g1_i1	-1.316153686	9.603882222	1.32E-08	1.12E-07	sepF
c662_g1_i1	1.761605026	7.77372243	7.30E-07	5.07E-06	serS
c213_g1_i1	4.155364902	5.90122239	3.33E-06	2.13E-05	sle1
c732_g1_i1	1.298508234	11.59171952	2.79E-09	2.60E-08	smpB
c2527_g1_i1	-1.855697227	6.600135491	1.00E-06	6.89E-06	sodM
c528_g1_i1	4.380375397	7.466984534	1.20E-16	2.25E-15	spa
c435_g1_i1	5.072547042	6.677303273	1.85E-11	2.18E-10	spa
c720_g1_i1	-3.620497565	7.390716206	9.45E-28	3.69E-26	ssaA1
c2374_g1_i1	-1.477957574	11.15594089	7.53E-12	9.22E-11	sucDC
c198_g1_i1	-1.115690073	8.019376964	6.95E-05	0.000378675	tagH
c1088_g1_i1	1.73062226	7.531570584	3.82E-06	2.42E-05	tagX
c305_g2_i1	2.048343851	6.612089996	6.14E-05	0.000337006	tcaA
c355_g1_i1	-12.68405425	6.643933196	1.62E-28	6.84E-27	tet
c355_g2_i1	-9.901073368	1.83152842	2.86E-05	0.000163099	tet
c1485_g1_i1	9.449809667	4.631638837	0.000184687	0.000938865	thiI
c405_g1_i1	-1.814318843	8.927090872	2.23E-13	3.28E-12	tig
c819_g1_i1	-10.3157336	2.403054939	1.36E-06	9.16E-06	tnp
c345_g1_i1	10.81445233	5.801177354	2.17E-09	2.07E-08	tnp
c469_g1_i1	4.357309909	6.071377639	3.32E-07	2.39E-06	tnpR
c546_g1_i1	-1.976619698	7.328267364	6.83E-10	7.00E-09	topA/trmFO
c374_g1_i1	-1.796598342	10.46680743	7.16E-16	1.28E-14	tpiA
c719_g4_i1	-1.820811375	6.438547278	3.58E-06	2.28E-05	trmD/rpsP
c616_g1_i1	-2.852241072	7.667724433	6.52E-21	1.55E-19	truB
c698_g1_i1	-1.39312826	9.057384953	1.01E-08	8.85E-08	trxB
c448_g1_i1	2.155552958	9.392947398	2.01E-15	3.40E-14	tyrA
c399_g1_i1	2.085021626	9.512496368	4.31E-15	7.02E-14	uvrBA
c706_g1_i1	-1.66072745	8.432551751	2.38E-10	2.53E-09	valS
c1074_g1_i1	3.459520516	6.685813464	1.19E-08	1.02E-07	vraAC
c2392_g1_i1	2.568405732	10.00151065	7.18E-23	2.01E-21	vraSR
c447_g1_i1	4.93898538	12.90967156	1.41E-82	4.15E-80	vraX
c512_g1_i1	-1.370395537	8.146579329	5.97E-07	4.18E-06	xerD
c1764_g1_i1	-1.448656652	6.769119397	6.94E-05	0.000378675	yidC
c33_g1_i1	-20.2042331	14.66080308	1.05E-259	2.17E-256	
c2139_g1_i1	-14.03350548	8.311070885	8.70E-67	1.39E-64	
c1664_g1_i1	-12.18937924	5.909431821	7.44E-21	1.73E-19	
c1514_g1_i1	-11.31516751	4.294948515	3.33E-12	4.21E-11	
c1852_g1_i1	-11.25379158	4.167487672	9.60E-12	1.17E-10	
c2423_g1_i2	-11.1896881	4.033874884	2.80E-11	3.23E-10	
c1856_g1_i1	-10.90031873	3.439471201	1.30E-09	1.27E-08	
c1808_g1_i1	-10.90031873	3.439471201	1.30E-09	1.27E-08	
c2511_g1_i1	-10.80629907	7.28993765	3.15E-39	1.86E-37	
c162_g2_i1	-10.63743528	2.939128239	7.04E-08	5.56E-07	
c1221_g1_i1	-10.3157336	2.403054939	1.36E-06	9.16E-06	
c2553_g1_i1	-10.19030562	2.216959066	2.48E-06	1.63E-05	
c2285_g1_i1	-10.05292557	2.026679388	8.35E-06	5.05E-05	
c1910_g1_i1	-10.05292557	2.026679388	8.35E-06	5.05E-05	
c162_g1_i1	-10.05292557	2.026679388	8.35E-06	5.05E-05	
c1496_g1_i1	-9.901073368	1.83152842	2.86E-05	0.000163099	
c2645_g1_i1	-9.731336964	1.630495967	9.96E-05	0.000525784	
c1266_g1_i1	-8.451461606	3.325025939	3.99E-09	3.66E-08	
c550_g1_i2	-7.74422573	5.933364223	3.17E-21	7.70E-20	
c400_g2_i1	-7.211825488	2.720393481	2.27E-07	1.67E-06	

c2225_g1_i1	-6.971007366	2.377431603	2.48E-06	1.63E-05
c414_g1_i1	-6.497746841	3.153652276	2.21E-08	1.82E-07
c279_g1_i1	-6.398275859	2.998407498	7.04E-08	5.56E-07
c375_g1_i1	-6.281174254	3.808216673	2.46E-10	2.60E-09
c722_g1_i1	-5.796805119	19.19469839	8.07E-118	4.18E-115
c600_g1_i1	-5.404201103	2.852048335	4.11E-07	2.94E-06
c691_g2_i1	-4.668842943	6.777006946	1.95E-31	8.97E-30
c313_g1_i1	-4.202081511	5.264646224	8.44E-15	1.33E-13
c1380_g1_i1	-3.610268892	8.758025883	9.85E-42	6.18E-40
c0_g2_i1	-3.328383195	7.569978159	3.64E-26	1.30E-24
c202_g2_i1	-3.288994751	8.990243833	1.46E-37	7.77E-36
c734_g2_i1	-3.285237417	16.71740574	5.00E-49	3.83E-47
c2006_g1_i1	-3.198434022	12.42217774	2.59E-46	1.85E-44
c202_g1_i1	-3.198126363	8.231565169	2.54E-30	1.12E-28
c564_g1_i1	-3.072326774	8.083767225	3.83E-27	1.44E-25
c498_g1_i1	-3.050920008	4.262024853	4.85E-06	3.02E-05
c1978_g1_i1	-3.041502295	3.83267745	5.25E-05	0.000290664
c536_g1_i1	-2.897792147	7.422087161	1.18E-19	2.57E-18
c750_g1_i1	-2.803321706	4.257975744	3.27E-05	0.000184365
c500_g3_i1	-2.715276773	4.560734921	1.35E-05	7.94E-05
c130_g1_i1	-2.538728652	8.315666399	5.34E-21	1.29E-19
c93_g1_i1	-2.534495754	8.518490377	6.78E-22	1.75E-20
c431_g1_i1	-2.488938369	8.033679138	1.17E-18	2.34E-17
c278_g1_i1	-2.47500247	7.513088019	1.42E-15	2.45E-14
c2464_g1_i1	-2.43772394	9.058274311	5.54E-23	1.57E-21
c2007_g1_i1	-2.400016041	8.390837136	2.35E-19	4.96E-18
c0_g1_i1	-2.361976688	9.426226216	4.87E-23	1.40E-21
c2027_g1_i1	-2.355499624	10.87766506	5.05E-26	1.74E-24
c1434_g1_i1	-2.351689163	7.84336722	5.52E-16	9.92E-15
c675_g3_i1	-2.333627376	8.789149042	3.25E-20	7.24E-19
c1439_g1_i1	-2.323900602	9.698279478	3.53E-23	1.03E-21
c2427_g1_i1	-2.316564694	9.029692166	7.24E-21	1.70E-19
c721_g1_i1	-2.308232778	7.919902792	8.51E-16	1.51E-14
c631_g1_i1	-2.256774927	7.11043376	1.87E-11	2.20E-10
c115_g1_i1	-2.17520225	8.900206563	2.56E-18	5.04E-17
c481_g1_i1	-2.125685993	7.980893639	5.43E-14	8.26E-13
c1981_g1_i1	-2.069197616	5.772735218	8.09E-06	4.94E-05
c472_g2_i1	-2.045883453	7.496902518	3.70E-11	4.20E-10
c86_g2_i1	-2.042361017	10.30297192	1.34E-19	2.88E-18
c537_g1_i1	-2.021143765	6.618936735	8.35E-08	6.45E-07
c2043_g1_i1	-1.969210658	6.229886479	2.54E-06	1.66E-05
c603_g1_i1	-1.968199511	5.884728191	1.42E-05	8.35E-05
c1388_g1_i1	-1.965104445	8.612024018	2.27E-14	3.56E-13
c657_g2_i1	-1.952642104	7.059989652	1.07E-08	9.42E-08
c1502_g1_i1	-1.922463732	5.550968174	0.000106442	0.000554732
c2045_g1_i1	-1.910738435	5.634031678	0.000102124	0.000533574
c717_g1_i1	-1.90513788	9.766892165	1.71E-16	3.17E-15
c35_g1_i1	-1.893050836	7.669975414	2.12E-10	2.28E-09
c625_g1_i1	-1.87027986	8.213313479	5.48E-12	6.75E-11
c836_g1_i1	-1.866692435	9.265791563	7.05E-15	1.12E-13
c247_g1_i1	-1.865029592	9.989109273	2.78E-16	5.09E-15
c118_g1_i1	-1.825349198	6.61211254	1.24E-06	8.41E-06
c2036_g1_i1	-1.823197894	6.527747795	2.53E-06	1.65E-05

c2421_g1_i1	-1.700753159	6.662335168	5.48E-06	3.40E-05
c346_g1_i1	-1.699332929	6.443181898	1.97E-05	0.000114773
c2493_g1_i1	-1.691173617	6.706644612	3.90E-06	2.45E-05
c415_g1_i1	-1.673108368	6.023426963	0.000145525	0.000747125
c1430_g1_i1	-1.659503042	7.408896149	1.45E-07	1.09E-06
c1395_g1_i1	-1.654182164	7.64248067	3.13E-08	2.54E-07
c759_g1_i1	-1.631918876	9.557991608	2.69E-12	3.43E-11
c511_g1_i1	-1.599332784	7.126402764	1.72E-06	1.15E-05
c544_g1_i1	-1.598430379	7.244595545	9.12E-07	6.29E-06
c565_g1_i1	-1.573614798	10.18100803	2.41E-12	3.12E-11
c696_g2_i1	-1.563827561	8.047993715	1.85E-08	1.54E-07
c2343_g1_i1	-1.41730702	7.661516723	2.35E-06	1.56E-05
c578_g1_i1	-1.380104732	9.035782555	1.57E-08	1.32E-07
c712_g1_i1	-1.341325753	10.4993491	1.06E-09	1.06E-08
c246_g1_i1	-1.299270854	8.85833992	1.75E-07	1.30E-06
c32_g1_i1	-1.297874769	11.39607297	1.34E-09	1.31E-08
c2386_g1_i1	-1.289166896	8.127113758	2.78E-06	1.80E-05
c508_g2_i1	-1.285761913	8.911752503	1.98E-07	1.46E-06
c426_g1_i1	-1.280901115	8.941928311	1.96E-07	1.46E-06
c810_g1_i1	-1.258872795	7.560486645	4.08E-05	0.000226793
c2571_g1_i1	-1.187566315	8.564147687	4.00E-06	2.51E-05
c2413_g1_i1	-1.161815817	8.300375178	1.47E-05	8.59E-05
c433_g1_i1	-1.126651305	11.89288222	1.01E-07	7.78E-07
c253_g1_i1	-1.111750208	10.31657217	5.42E-07	3.83E-06
c621_g1_i1	-1.111035783	7.69233585	0.000190921	0.000968173
c369_g1_i1	-1.107025068	8.436533967	2.64E-05	0.000151902
c1812_g1_i1	-1.025601045	9.444578728	1.28E-05	7.57E-05
c498_g3_i1	1.063087314	8.770495057	0.000101886	0.000533574
c2106_g1_i1	1.114528902	9.156671465	1.77E-05	0.000103352
c314_g1_i1	1.328487906	7.575512469	0.000197981	0.000999081
c1068_g1_i1	1.342899094	9.081145741	4.68E-07	3.32E-06
c347_g1_i1	1.380051021	9.251530602	1.21E-07	9.16E-07
c2436_g1_i1	1.472954614	7.961425671	7.86E-06	4.81E-05
c1196_g1_i1	1.55615908	7.547651257	2.15E-05	0.000124541
c2434_g1_i1	1.574301765	7.411926459	3.41E-05	0.000191918
c2048_g1_i1	1.578747445	9.276474588	2.20E-09	2.09E-08
c2370_g1_i1	1.614749234	7.084712781	0.000131267	0.000677286
c1714_g1_i1	1.640083828	8.319448958	1.34E-07	1.01E-06
c300_g1_i1	1.647953167	10.8239779	6.56E-13	8.99E-12
c701_g1_i1	1.674935286	7.707162762	2.52E-06	1.65E-05
c1080_g1_i1	1.737565082	9.263691805	7.88E-11	8.74E-10
c731_g11_i1	1.75017288	7.287571587	1.22E-05	7.26E-05
c137_g1_i1	1.908246104	9.026211971	1.07E-11	1.28E-10
c1401_g1_i1	1.91977175	9.450851637	4.20E-13	5.95E-12
c1443_g1_i1	1.944918591	7.981969827	1.59E-08	1.33E-07
c1522_g1_i1	1.951686588	8.167857974	3.33E-09	3.09E-08
c502_g1_i1	1.994738412	6.569440487	0.000114842	0.000596783
c731_g2_i2	2.022659682	8.699092644	1.31E-11	1.57E-10
c632_g2_i1	2.072187914	7.798389785	1.30E-08	1.11E-07
c59_g1_i1	2.179827018	7.043718106	1.55E-06	1.04E-05
c1715_g1_i1	2.313885704	11.63196281	3.10E-24	1.00E-22
c430_g1_i1	2.355553578	6.659435448	8.69E-06	5.24E-05
c592_g1_i1	2.361540747	9.560721768	2.50E-18	4.97E-17

c387_g1_i1	2.374533176	6.440743397	3.01E-05	0.000170873
c221_g1_i1	2.463486717	6.746774474	2.14E-06	1.43E-05
c460_g1_i1	2.532434068	6.284320257	3.42E-05	0.000191918
c595_g1_i1	2.580170687	9.795424922	5.51E-22	1.44E-20
c2364_g1_i1	2.582861336	6.843794038	4.05E-07	2.91E-06
c412_g1_i1	2.62681063	8.714034818	1.12E-16	2.13E-15
c8_g1_i1	2.821749734	6.157082345	2.57E-05	0.000148301
c2381_g1_i1	2.94448268	9.851700185	6.69E-27	2.47E-25
c717_g3_i1	2.95120561	6.263835925	6.45E-06	3.98E-05
c1409_g1_i1	3.031228697	9.576890282	3.57E-26	1.29E-24
c2008_g1_i1	3.071357095	5.854088438	8.65E-05	0.000461005
c475_g1_i1	3.165079633	7.32406518	2.45E-11	2.85E-10
c149_g1_i1	3.202386171	5.963035022	2.79E-05	0.00015989
c614_g1_i1	3.247564076	6.87335911	3.72E-09	3.43E-08
c404_g1_i1	3.43542457	6.665689572	1.33E-08	1.12E-07
c691_g1_i1	3.486209791	7.358841025	9.54E-13	1.27E-11
c44_g1_i1	3.496590749	6.716799694	4.80E-09	4.35E-08
c603_g2_i1	3.631484595	6.829840286	8.79E-10	8.83E-09
c634_g1_i1	3.701404653	10.00203095	1.14E-37	6.21E-36
c797_g1_i1	3.702450709	8.581588412	4.66E-24	1.46E-22
c1382_g1_i1	3.901874451	5.687827594	2.93E-05	0.000166751
c82_g1_i1	4.042226189	6.665797998	6.00E-10	6.20E-09
c2061_g1_i1	4.07638191	9.986424808	4.79E-42	3.10E-40
c190_g1_i1	4.149112062	6.756099651	1.37E-10	1.50E-09
c2423_g1_i1	4.152219997	5.898574064	3.33E-06	2.13E-05
c568_g2_i1	4.212925584	8.605704611	1.43E-27	5.49E-26
c728_g2_i1	4.395047859	6.103202426	2.79E-07	2.03E-06
c1763_g1_i1	4.485287499	6.179348427	7.45E-08	5.79E-07
c419_g2_i1	4.54405716	6.228979084	4.07E-08	3.27E-07
c668_g1_i1	4.663729191	6.330154899	7.38E-09	6.55E-08
c251_g2_i1	4.707236914	6.366980496	5.28E-09	4.75E-08
c2357_g1_i1	4.739027734	6.39390436	2.72E-09	2.55E-08
c1730_g1_i1	5.115163571	6.713657556	5.47E-12	6.75E-11
c1507_g1_i1	5.333989944	9.793353076	1.40E-51	1.11E-49
c647_g2_i1	5.378671514	6.939299381	5.26E-14	8.05E-13
c1983_g1_i1	5.54016398	7.078393712	1.49E-15	2.54E-14
c58_g1_i1	5.556908532	7.092854177	8.69E-16	1.52E-14
c2358_g1_i1	5.898831031	10.16418721	9.71E-62	1.26E-59
c422_g1_i1	6.034417684	7.508575159	8.07E-21	1.86E-19
c1698_g1_i1	6.138174354	9.739382346	9.35E-56	9.21E-54
c2345_g1_i1	6.367341957	9.648667784	3.42E-55	3.22E-53
c409_g2_i1	6.575081989	7.98798278	5.16E-28	2.09E-26
c673_g1_i1	6.668638869	10.7165843	1.87E-77	3.88E-75
c664_g2_i1	6.83649051	9.143976402	1.22E-47	9.01E-46
c1087_g1_i1	7.069631066	9.919413662	2.24E-63	3.10E-61
c469_g2_i1	7.282850629	9.564243924	7.02E-57	7.65E-55
c69_g1_i1	7.516559136	10.34875159	3.69E-74	6.95E-72
c494_g1_i1	7.681297557	12.47404372	9.84E-121	6.78E-118
c207_g1_i1	9.388498815	4.578604732	0.00016937	0.000867395
c367_g1_i1	9.46685891	4.646377523	0.000184687	0.000938865
c344_g1_i1	9.524992893	4.696603688	8.39E-05	0.000448565
c563_g2_i1	9.549207801	4.717511331	8.39E-05	0.000448565
c745_g1_i1	9.549207801	4.717511331	8.39E-05	0.000448565

c349_g3_i1	9.580874799	4.744841431	5.65E-05	0.00031147
c370_g1_i1	9.798515715	4.932319403	9.16E-06	5.51E-05
c1697_g1_i1	9.877141668	4.999900701	1.06E-05	6.36E-05
c1115_g1_i1	9.896146396	5.016224567	7.29E-06	4.48E-05
c704_g1_i3	10.05114166	5.149200379	2.80E-06	1.81E-05
c726_g2_i1	10.20131192	5.277793886	7.87E-07	5.45E-06
c344_g3_i1	10.3829381	5.433054461	1.17E-07	8.91E-07
c383_g1_i1	10.47007317	5.507455556	7.31E-08	5.73E-07
c1116_g1_i1	10.54824466	5.574165958	1.91E-08	1.58E-07
c349_g1_i1	10.56417561	5.587757545	3.33E-08	2.68E-07
c383_g2_i1	10.5720756	5.594497054	3.33E-08	2.68E-07
c486_g2_i1	10.67836024	5.685146154	1.13E-08	9.78E-08
c1109_g1_i1	10.68566059	5.691371259	1.13E-08	9.78E-08
c771_g1_i1	10.70375147	5.706797026	8.25E-09	7.29E-08
c2399_g1_i1	10.98095425	5.943129449	1.80E-10	1.96E-09
c697_g2_i1	11.07011942	6.019169835	7.28E-11	8.14E-10
c468_g2_i1	11.09500646	6.040398641	4.04E-11	4.57E-10
c1704_g1_i1	11.09774537	6.04273511	3.02E-11	3.47E-10
c275_g1_i1	11.14352849	6.081796272	3.05E-11	3.48E-10
c427_g1_i1	11.19559438	6.126231163	9.70E-12	1.17E-10
c143_g1_i1	11.21590576	6.143569997	5.48E-12	6.75E-11
c868_g1_i1	11.35747391	6.264502623	1.14E-12	1.49E-11
c697_g3_i1	11.35747391	6.264502623	1.14E-12	1.49E-11
c574_g2_i1	11.40247456	6.302979303	3.87E-13	5.55E-12
c37_g2_i1	11.44826159	6.342148817	3.19E-13	4.62E-12
c607_g2_i1	11.53569406	6.417008282	7.24E-14	1.08E-12
c2349_g1_i1	11.5656788	6.442702022	3.35E-14	5.21E-13
c1106_g1_i1	11.7300448	6.583761989	2.15E-15	3.58E-14
c607_g1_i1	11.95665759	6.778947464	3.25E-17	6.35E-16
c1442_g1_i1	12.14320959	6.940363113	5.08E-19	1.06E-17
c2051_g1_i1	12.31309915	7.088029369	9.23E-21	2.10E-19
c2416_g1_i1	12.5802051	7.321649336	2.30E-23	6.90E-22
c731_g4_i1	13.2607571	7.926203027	1.00E-31	4.72E-30
c37_g1_i1	13.7380891	8.359063575	7.99E-39	4.59E-37

Supplementary Table S5. Differentially expressed genes in 2VR compared with 236C05

ID	log2(fold_change)	logCPM	P_Value	FDR	gene name
c637_g2_i1	-2.280224409	11.5622365	1.47E-25	5.98E-24	acnA
c139_g1_i1	-1.420702373	9.980088336	3.10E-10	3.89E-09	acyP
c794_g1_i1	14.00936699	7.985746466	6.27E-34	3.59E-32	adh
c556_g1_i1	7.154388746	13.68355353	4.55E-134	4.82E-131	agrA
c608_g1_i1	-2.728254861	12.6204787	1.84E-35	1.25E-33	ahpCF
c526_g1_i1	3.16869596	8.447040954	2.62E-19	6.53E-18	araB
c477_g2_i1	4.387995795	6.098816158	1.85E-07	1.64E-06	arcAB
c2340_g1_i1	3.111155775	9.969244041	5.76E-30	2.91E-28	argGH
c674_g2_i1	12.20937839	6.410874834	1.15E-14	2.08E-13	arsRBC
c1381_g1_i1	1.91776042	10.83513041	1.46E-16	3.05E-15	asp23
c2371_g2_i1	1.051665205	10.06112815	7.60E-06	5.16E-05	atl
c2371_g1_i1	4.21943061	7.992809042	2.24E-21	6.42E-20	atl
c1780_g1_i1	4.433222813	6.136990322	1.08E-07	9.70E-07	bbp
c147_g1_i1	3.379008995	6.113358009	3.80E-06	2.71E-05	betA
c1705_g1_i1	-13.50544698	6.81011546	2.16E-32	1.21E-30	blaZ
c1077_g1_i1	-1.512369057	10.22304836	1.27E-11	1.79E-10	butA
c434_g1_i1	1.516239934	9.35531111	5.19E-09	5.53E-08	cadAC
c1520_g1_i1	-11.41838728	2.640547483	7.04E-09	7.31E-08	cadC
c552_g4_i1	1.738736902	7.690838495	1.31E-06	1.00E-05	cap5A
c1386_g1_i1	-1.817021579	8.901586451	1.68E-13	2.78E-12	cdr
c40_g1_i1	2.280316559	7.887616421	3.48E-10	4.29E-09	cidB
c710_g2_i2	-12.22551628	4.598128477	1.21E-14	2.17E-13	clfA
c710_g2_i1	10.4232164	4.88174866	1.03E-05	6.83E-05	clfA
c723_g3_i1	-1.095739165	8.168320557	5.88E-05	0.000335798	clfB
c743_g1_i1	-1.019694508	13.17284034	9.85E-07	7.73E-06	clpB
c15_g2_i1	-2.301196929	13.0451708	1.41E-26	6.65E-25	clpC
c15_g1_i1	-1.938761694	11.39705663	3.68E-19	9.06E-18	clpC
c1083_g1_i1	12.23289283	6.431012502	5.94E-15	1.10E-13	clpL
c602_g1_i1	-1.100889107	9.791013006	1.37E-06	1.03E-05	clpP
c1699_g1_i1	-2.252237164	9.311878638	5.14E-21	1.41E-19	clpX
c49_g1_i1	2.546809442	6.584646093	4.53E-06	3.19E-05	cls1
c630_g1_i1	1.781601745	6.870831277	9.15E-05	0.000508676	cls2
c589_g1_i1	4.404311542	6.11258611	1.94E-07	1.71E-06	cmk
c690_g1_i1	-2.962254321	7.092522551	4.87E-18	1.09E-16	cna
c731_g1_i1	-1.730172704	8.152590335	2.39E-10	3.01E-09	copA
c350_g1_i1	5.562249609	7.098811887	3.12E-16	6.41E-15	copB
c731_g5_i1	-2.019913843	5.395146551	7.68E-05	0.000430545	copZ
c2044_g1_i1	-1.826618773	9.626396445	3.56E-15	6.73E-14	cspA
c731_g2_i1	1.454800959	7.899984874	1.25E-05	8.23E-05	ctaA
c733_g2_i1	1.560078902	7.144994019	0.000137261	0.000739742	dapABH
c90_g1_i1	11.97994907	6.214677544	2.77E-13	4.51E-12	dapE
c768_g1_i1	1.673757324	8.548162739	1.73E-08	1.71E-07	ddl
c517_g1_i1	2.472013197	9.879193404	4.28E-21	1.21E-19	dinB/dagK
c1691_g1_i1	1.419272323	9.341034095	4.13E-08	3.94E-07	dltAC
c449_g1_i1	-1.202236284	8.922916169	9.85E-07	7.73E-06	dnaNA
c105_g1_i1	10.14394815	4.639569136	5.09E-05	0.000297673	ebhA
c1831_g1_i1	-8.528747585	1.710699008	1.36E-06	1.02E-05	ermA
c2020_g1_i1	1.529500119	7.600231323	2.11E-05	0.000131623	esxA
c713_g1_i1	-1.040745414	9.019511062	1.96E-05	0.000123423	fabDG
c524_g1_i1	4.674284312	7.725864721	3.26E-20	8.42E-19	fbp
c1749_g1_i1	-1.962163117	9.6415933	2.96E-17	6.41E-16	fda

c577_g3_i1	-1.967473954	7.167222469	2.63E-09	2.95E-08	fdhD
c856_g1_i1	3.918415628	6.565034323	5.04E-09	5.42E-08	femAB
c1996_g1_i1	-1.018313589	9.229737373	1.97E-05	0.000123508	femBA
c1139_g1_i1	12.13513114	6.347328971	4.07E-14	7.13E-13	fmtA
c2171_g1_i1	-1.146932348	8.688498096	5.87E-06	4.09E-05	ftnA
c1420_g1_i1	1.878869361	8.223453635	5.33E-09	5.64E-08	fumC
c598_g1_i1	-2.52878864	6.810835911	2.75E-12	4.09E-11	fur
c2010_g1_i1	4.96807588	10.49428718	6.24E-59	8.26E-57	gapA2
c189_g1_i2	-5.449822536	3.416938954	1.42E-10	1.82E-09	glcB
c2386_g1_i1	-1.167379109	8.173481352	1.72E-05	0.000109373	glcT
c655_g1_i1	-1.82705892	7.128697395	3.95E-08	3.78E-07	glcU
c136_g2_i1	2.110273834	8.457192154	1.88E-11	2.62E-10	glmS
c136_g1_i1	4.506903435	7.580242762	2.53E-18	5.69E-17	glmS
c291_g1_i1	2.937275053	6.256062267	7.57E-06	5.16E-05	glpT
c1097_g1_i1	-1.034492254	10.44412016	2.50E-06	1.81E-05	gnd
c238_g1_i1	-1.902315319	14.89524958	2.58E-19	6.50E-18	gpsB
c557_g1_i1	2.391053153	7.480421463	5.19E-09	5.53E-08	graRS
c670_g2_i1	-1.665572616	9.618836062	6.20E-13	9.88E-12	greA
c594_g1_i1	2.643910487	10.09117281	2.47E-24	9.16E-23	groLS
c731_g2_i8	10.16982733	4.662072159	5.09E-05	0.000297673	guaBA
c779_g1_i1	2.214443812	6.921999048	2.65E-06	1.91E-05	guaC
c191_g1_i1	7.086520009	12.75815013	6.60E-120	2.79E-117	hld
c68_g1_i1	-1.721664566	9.814148102	5.53E-14	9.60E-13	hpt
c479_g2_i1	-1.246464153	13.43612281	2.42E-09	2.73E-08	hrcA/dnaK
c342_g1_i1	-2.173011437	7.012954333	1.67E-10	2.12E-09	hslO
c561_g1_i1	2.108396637	6.464368601	0.000100547	0.000551707	hutG
c701_g2_i1	3.011415408	6.96620821	5.75E-09	6.03E-08	hutIU
c324_g1_i1	-1.366177592	11.78002919	1.33E-10	1.72E-09	icd
c545_g1_i1	2.606395703	7.655796644	7.19E-11	9.57E-10	ilvE
c1991_g1_i1	-1.720183872	9.78562847	6.37E-14	1.10E-12	isaA
c2342_g1_i1	-2.319579905	11.7801165	1.82E-26	8.36E-25	katA
c474_g2_i1	10.64975617	5.077204465	2.37E-06	1.72E-05	lacEG
c2112_g1_i1	3.640470468	8.53142114	2.50E-23	8.42E-22	ldhA
c1724_g1_i1	1.153370406	9.187266307	8.51E-06	5.74E-05	lexA
c243_g1_i1	5.09468728	8.096226718	5.72E-26	2.47E-24	lip2
c654_g1_i1	-1.003192869	9.652121236	1.34E-05	8.74E-05	lipA
c54_g1_i1	-1.93250499	9.363065088	3.40E-16	6.93E-15	lytH
c567_g1_i1	-2.617558003	7.295830803	4.53E-16	9.04E-15	lytM
c1817_g1_i1	3.38523657	6.627827981	2.28E-08	2.21E-07	map
c1103_g1_i1	1.554578307	7.477293041	2.93E-05	0.000177351	metG
c543_g2_i1	10.8741207	5.27001813	3.23E-07	2.79E-06	metN1
c1729_g1_i1	3.175758553	9.065979337	3.50E-24	1.28E-22	mgt
c553_g1_i1	-1.142429158	9.603085076	7.50E-07	6.07E-06	mnhA1
c4_g1_i1	4.512372109	7.068484624	7.50E-14	1.27E-12	mnhD1
c695_g1_i1	-1.735925639	6.974177291	5.50E-07	4.59E-06	mntH
c687_g2_i1	-1.622854993	8.517761316	3.36E-10	4.19E-09	moaADE
c1447_g1_i1	2.931958691	8.040762404	6.05E-15	1.11E-13	mprF
c1805_g1_i1	1.05083826	8.668722421	0.000150819	0.000804623	mqo1
c1737_g1_i1	2.2169504	7.340328276	9.61E-08	8.73E-07	msrR
c717_g1_i1	-1.183308771	9.971232546	1.55E-07	1.38E-06	murC
c547_g1_i1	1.790567187	7.58165884	1.51E-06	1.13E-05	murE
c170_g1_i1	3.133259712	9.029203307	1.73E-23	6.00E-22	murQ
c677_g1_i1	-1.933379365	10.91171297	1.01E-18	2.38E-17	mutS2/trxA
c1725_g1_i1	1.90804367	7.506053548	6.46E-07	5.32E-06	nadE

c2121_g1_i1	-1.546484022	7.422412789	7.14E-07	5.85E-06	nfrA
c656_g1_i1	2.051486887	7.86251382	8.70E-09	8.99E-08	norA
c1394_g1_i1	3.599828989	11.41788606	9.92E-47	9.55E-45	nrdI/nrdF
c647_g1_i1	6.009952058	7.488127404	5.96E-21	1.62E-19	nreCB
c1253_g1_i1	-13.41799189	6.688779098	1.69E-30	8.97E-29	pbp
c817_g1_i1	-10.63690088	8.691416639	1.16E-86	3.08E-84	pbp
c921_g1_i1	-10.56058976	5.922037222	2.54E-22	8.03E-21	pbp
c1469_g1_i1	-9.238942826	3.077775901	4.27E-10	5.17E-09	pbp
c193_g1_i1	1.423974942	10.5679011	6.38E-10	7.55E-09	pckA
c442_g1_i1	-3.252860209	12.41208137	1.21E-47	1.22E-45	pdhBCD
c715_g2_i3	1.068022815	9.283662669	2.70E-05	0.000164271	pdp/deoC2
c2336_g1_i1	5.648505761	10.49602859	5.49E-66	8.31E-64	pdxS
c1438_g1_i1	4.923036491	9.921669854	2.13E-50	2.25E-48	pflAB
c726_g1_i1	8.129015376	9.420258528	5.92E-57	7.37E-55	pre
c2430_g1_i1	3.261474165	8.706763138	5.12E-22	1.51E-20	prsA
c1992_g1_i1	1.995485617	7.164245808	3.16E-06	2.26E-05	purA
c394_g1_i1	3.697807923	10.42813755	1.63E-41	1.33E-39	purQLME
c671_g1_i1	-2.216551529	8.732549502	1.96E-18	4.47E-17	queA/tgt
c2076_g1_i1	10.72848556	5.14494284	6.39E-07	5.29E-06	queF
c26_g1_i1	2.113542697	9.585667041	1.04E-15	2.02E-14	recA
c699_g1_i1	2.241694436	8.284495589	1.06E-11	1.50E-10	ribBA
c642_g1_i1	-3.535683425	9.426511122	4.74E-46	4.37E-44	ribU
c713_g2_i1	-1.055700927	8.457597767	5.60E-05	0.000321325	rnc
c679_g1_i1	-1.058514816	8.524113547	4.14E-05	0.000245031	rnpA
c1703_g1_i1	-1.112000268	11.05195709	2.38E-07	2.08E-06	rny
c585_g1_i1	-1.241008481	13.17206259	2.98E-09	3.31E-08	rplV
c1688_g1_i1	-1.374385737	8.794593584	3.39E-08	3.26E-07	rpmF
c115_g1_i1	-1.228859253	9.16331669	2.99E-07	2.60E-06	rpmG3
c716_g3_i1	-1.173614772	7.901243229	4.03E-05	0.000240704	rpmI
c1071_g1_i1	-1.206883124	8.666778703	1.87E-06	1.38E-05	rpsB
c228_g1_i1	-1.01699066	12.52439975	1.20E-06	9.35E-06	rpsL
c1183_g1_i1	-2.703312675	4.950615064	1.23E-06	9.53E-06	rpsN
c616_g2_i1	-2.390780546	9.86260661	5.47E-25	2.07E-23	rpsO
c46_g1_i1	-1.925134353	8.4078322	2.10E-13	3.45E-12	rpsT
c1378_g1_i1	1.56545527	9.270112324	2.87E-09	3.20E-08	rsmH
c731_g6_i1	3.823686519	7.650269029	2.03E-16	4.21E-15	sarZ
c723_g2_i1	10.32989013	4.800974888	1.33E-05	8.71E-05	sdrC
c723_g1_i1	10.23010657	4.714438424	2.47E-05	0.00015104	sdrD
c723_g5_i1	11.11960497	5.480274755	2.07E-08	2.04E-07	sdrD
c1719_g1_i1	1.026061013	9.749952811	2.19E-05	0.000136144	secA2
c1411_g1_i1	2.908059035	9.261358461	7.67E-23	2.50E-21	sepA
c534_g1_i1	-1.113954419	9.673110962	1.23E-06	9.53E-06	sepF/ylmD
c213_g1_i1	5.557880548	7.095040283	3.61E-16	7.29E-15	sle1
c732_g1_i1	2.148414223	12.24598286	3.22E-22	9.87E-21	smpB
c2527_g1_i1	-2.039647797	6.520007731	9.59E-08	8.73E-07	sodM
c528_g1_i1	2.777118316	6.123863184	4.05E-05	0.00024075	spa
c435_g1_i1	3.740360468	5.552639817	0.000104757	0.000573322	spa
c683_g3_i1	1.722150561	7.269616088	1.65E-05	0.000105374	spsAB
c720_g1_i1	-3.360931174	7.421567656	1.18E-25	4.89E-24	ssaA1
c720_g2_i1	1.717327861	9.274932711	1.15E-10	1.51E-09	ssaA2
c305_g2_i1	2.372110419	6.875732264	1.29E-06	9.94E-06	tcaA
c355_g1_i1	-9.640202963	6.628602686	6.74E-30	3.32E-28	tet
c705_g1_i1	10.13346509	4.630450045	7.28E-05	0.000410351	thiI
c405_g1_i1	-1.190421916	9.118023127	7.86E-07	6.31E-06	tig

c819_g1_i1	-8.654214585	1.908701701	4.11E-07	3.49E-06	tnp
c1172_g1_i1	-7.877108863	0.899260128	0.000187	0.000987696	tnp
c345_g1_i1	11.07373653	5.441035532	2.33E-08	2.26E-07	tnp
c469_g1_i1	4.187652075	5.929833667	1.87E-06	1.38E-05	tnpR
c705_g2_i1	-1.311802818	9.716039829	1.08E-08	1.10E-07	tpx/ackA
c1692_g1_i1	4.371493417	6.08489027	2.26E-07	1.98E-06	trpS
c616_g1_i1	-2.54868083	7.726475076	7.72E-18	1.70E-16	truB
c698_g1_i1	-1.302983625	9.088464104	7.16E-08	6.65E-07	trxB
c399_g1_i1	1.768514761	9.26766975	3.80E-11	5.20E-10	uvrAB
c706_g1_i1	-1.150086613	8.604011444	6.91E-06	4.78E-05	valS
c1074_g1_i1	3.145177204	6.428178124	5.95E-07	4.94E-06	vraAC
c2392_g1_i1	2.700434692	10.11711916	3.21E-25	1.24E-23	vraSR
c447_g1_i1	6.258852926	14.20101043	1.45E-120	7.69E-118	vraX
c512_g1_i1	-1.65760732	8.050247396	2.01E-09	2.28E-08	xerD
c2511_g1_i1	-13.86090054	7.279351935	2.31E-41	1.74E-39	
c33_g1_i1	-13.84734036	14.6643312	2.34E-270	4.96E-267	
c1514_g1_i1	-12.00317406	4.070875164	6.97E-13	1.08E-11	
c1808_g1_i1	-11.58825371	3.034449399	4.27E-10	5.17E-09	
c1221_g1_i1	-11.00352547	1.830415658	4.11E-07	3.49E-06	
c2225_g1_i1	-10.87805847	1.625462512	1.36E-06	1.02E-05	
c162_g1_i1	-10.7406316	1.418861894	4.54E-06	3.19E-05	
c1496_g1_i1	-10.58872219	1.209673337	1.54E-05	9.87E-05	
c2645_g1_i1	-10.41891429	0.996614761	5.33E-05	0.00030919	
c2139_g1_i1	-10.29683665	8.31081671	1.14E-73	2.19E-71	
c1856_g1_i1	-9.238942826	3.077775901	4.27E-10	5.17E-09	
c1664_g1_i1	-9.145493933	5.874805857	5.85E-22	1.70E-20	
c162_g2_i1	-8.976002128	2.49939348	1.25E-08	1.25E-07	
c2553_g1_i1	-8.528747585	1.710699008	1.36E-06	1.02E-05	
c2285_g1_i1	-8.391320716	1.51164439	4.54E-06	3.19E-05	
c1910_g1_i1	-8.391320716	1.51164439	4.54E-06	3.19E-05	
c1266_g1_i1	-8.305500625	2.933907672	1.30E-09	1.49E-08	
c355_g2_i1	-8.239411307	1.310791224	1.54E-05	9.87E-05	
c1852_g1_i1	-8.209799308	3.984619317	1.97E-12	2.96E-11	
c414_g1_i1	-7.59332352	2.609510234	1.25E-08	1.25E-07	
c550_g1_i2	-7.352232418	5.899975135	1.11E-22	3.57E-21	
c600_g1_i1	-6.883901075	2.116761179	2.27E-07	1.99E-06	
c279_g1_i1	-6.800959933	2.532213389	2.21E-08	2.16E-07	
c375_g1_i1	-6.275354849	3.548585139	4.79E-11	6.51E-10	
c400_g2_i1	-5.470779624	2.690988726	1.25E-08	1.25E-07	
c722_g1_i1	-5.364526225	19.20706078	2.52E-105	8.89E-103	
c691_g2_i1	-5.266707511	6.718107155	2.43E-34	1.47E-32	
c32_g1_i1	-4.46777024	10.95138078	1.07E-74	2.53E-72	
c734_g2_i1	-4.263063099	16.65317089	1.91E-74	4.04E-72	
c202_g1_i1	-3.740814848	8.166523496	3.73E-39	2.72E-37	
c202_g2_i1	-3.476425701	8.970845543	5.70E-42	4.83E-40	
c498_g1_i1	-3.378415671	3.996031646	1.33E-06	1.01E-05	
c625_g1_i1	-3.210242152	7.934203591	2.00E-28	9.64E-27	
c0_g2_i1	-3.061087404	7.608294466	1.67E-23	5.98E-22	
c313_g1_i1	-3.059647655	5.549647414	3.44E-10	4.26E-09	
c631_g1_i1	-3.003256331	6.913956279	2.47E-17	5.38E-16	
c2006_g1_i1	-2.992405688	12.44748702	2.02E-41	1.59E-39	
c0_g1_i1	-2.99091046	9.328554425	1.79E-34	1.15E-32	
c750_g1_i1	-2.901758431	4.123835061	1.98E-05	0.000123808	
c2423_g1_i2	-2.874927658	5.060635702	1.02E-07	9.26E-07	

c2036_g1_i1	-2.803762192	6.163070309	2.88E-11	3.96E-10
c93_g1_i1	-2.753634128	8.479333042	1.63E-25	6.53E-24
c481_g1_i1	-2.681690145	7.853226612	2.14E-20	5.67E-19
c431_g1_i1	-2.450000452	8.040806884	1.75E-18	4.03E-17
c564_g1_i1	-2.389485933	8.207200628	1.72E-18	4.00E-17
c759_g1_i1	-2.364753195	9.392905689	3.63E-23	1.20E-21
c118_g1_i1	-2.303929851	6.422848582	4.68E-09	5.06E-08
c536_g1_i1	-2.284990915	7.557114363	6.48E-14	1.11E-12
c128_g1_i1	-2.184629939	7.964867213	9.31E-15	1.70E-13
c278_g1_i1	-2.180891881	7.584877267	5.65E-13	9.13E-12
c346_g1_i1	-2.176755621	6.234974701	1.36E-07	1.22E-06
c2027_g1_i1	-2.129582905	10.92211998	3.36E-22	1.02E-20
c472_g2_i1	-2.120702647	7.472206476	7.57E-12	1.09E-10
c369_g1_i1	-2.11320009	8.119838944	1.38E-14	2.46E-13
c1439_g1_i1	-2.052539345	9.755124174	6.91E-19	1.66E-17
c565_g1_i1	-2.008069397	10.0781287	8.60E-19	2.05E-17
c810_g1_i1	-1.866781962	7.334949036	3.85E-09	4.23E-08
c2007_g1_i1	-1.859984478	8.519711114	5.77E-13	9.26E-12
c744_g1_i1	-1.823549777	10.81957782	7.99E-17	1.69E-15
c2005_g1_i1	-1.770946345	6.403074929	8.96E-06	6.01E-05
c675_g3_i1	-1.762735472	8.924862362	8.35E-13	1.27E-11
c1388_g1_i1	-1.723496085	8.677585207	9.86E-12	1.40E-10
c537_g1_i1	-1.661852485	6.751749205	4.55E-06	3.19E-05
c2427_g1_i1	-1.65313073	9.188649785	5.14E-12	7.51E-11
c364_g1_i1	-1.644098862	6.410874905	3.06E-05	0.00018467
c1434_g1_i1	-1.618442925	8.046484872	5.58E-09	5.88E-08
c588_g1_i1	-1.613037017	11.12716345	8.91E-14	1.50E-12
c712_g1_i1	-1.588216654	10.43264411	6.29E-13	9.94E-12
c836_g1_i1	-1.542031475	9.354720779	6.32E-11	8.47E-10
c700_g1_i1	-1.541270138	11.41600721	7.10E-13	1.09E-11
c737_g1_i1	-1.527038398	7.479623795	7.40E-07	6.01E-06
c2571_g1_i1	-1.488767257	8.461823212	1.06E-08	1.09E-07
c1380_g1_i1	-1.486901247	9.152800783	6.04E-10	7.19E-09
c247_g1_i1	-1.470346271	10.09426153	5.89E-11	7.95E-10
c265_g1_i1	-1.44302628	6.640756755	0.000123264	0.000667707
c2464_g1_i1	-1.434826476	9.303258513	1.38E-09	1.57E-08
c353_g1_i1	-1.423901955	7.805580741	9.52E-07	7.54E-06
c1810_g1_i1	-1.416949559	6.695660705	0.000141157	0.000758808
c2126_g1_i1	-1.372275322	7.538644719	7.07E-06	4.87E-05
c578_g1_i1	-1.368511156	9.041365189	1.66E-08	1.65E-07
c86_g2_i1	-1.353677031	10.47944467	7.36E-10	8.57E-09
c2354_g1_i1	-1.34323964	8.004919045	1.53E-06	1.14E-05
c264_g1_i1	-1.335011593	9.457246702	1.12E-08	1.14E-07
c2343_g1_i1	-1.233221641	7.731901365	2.89E-05	0.000175204
c1430_g1_i1	-1.227670032	7.572232917	5.41E-05	0.000312426
c709_g1_i1	-1.225383478	8.513830912	2.22E-06	1.61E-05
c544_g1_i1	-1.213588282	7.396267805	0.000120174	0.000652639
c721_g1_i1	-1.193373415	8.258949427	8.69E-06	5.85E-05
c1812_g1_i1	-1.160283973	9.399409709	7.71E-07	6.21E-06
c246_g1_i1	-1.109136781	8.926745623	6.69E-06	4.64E-05
c130_g1_i1	-1.095393292	8.719073117	1.37E-05	8.87E-05
c250_g1_i1	1.004431532	9.900040269	2.37E-05	0.00014561
c94_g1_i1	1.083646318	8.560880872	0.000129894	0.000701825
c1401_g1_i1	1.173768553	8.897380871	1.34E-05	8.74E-05

c1113_g1_i1	1.234707118	9.188865622	2.04E-06	1.49E-05
c680_g1_i1	1.319420576	7.949031067	4.67E-05	0.000275713
c1084_g1_i1	1.346941564	7.652958795	0.000115816	0.000630589
c592_g1_i1	1.353241521	8.780518947	1.18E-06	9.25E-06
c701_g1_i1	1.48699777	7.568166703	4.09E-05	0.000242405
c633_g1_i1	1.496589299	7.43336781	6.50E-05	0.000370005
c444_g1_i1	1.592281013	10.23492753	2.16E-11	2.99E-10
c508_g1_i1	1.62256774	7.452351601	1.80E-05	0.000114023
c612_g1_i1	1.658302409	8.248642491	1.35E-07	1.21E-06
c632_g2_i1	1.695900214	7.508580354	5.85E-06	4.09E-05
c1377_g1_i1	1.698837587	7.046988423	6.60E-05	0.000374987
c1480_g1_i1	1.704518844	7.733563025	1.64E-06	1.22E-05
c2444_g1_i1	1.736370805	7.182165391	2.39E-05	0.000146557
c137_g1_i1	1.736679235	8.897011446	7.32E-10	8.57E-09
c25_g1_i1	1.741683391	10.16081031	6.51E-13	1.02E-11
c2403_g1_i1	1.918674382	7.103712352	7.85E-06	5.31E-05
c731_g11_i1	1.933901677	7.434572602	8.05E-07	6.43E-06
c1696_g1_i1	1.934189592	7.526543005	4.64E-07	3.93E-06
c138_g1_i1	1.960993984	8.388613106	4.80E-10	5.78E-09
c1068_g1_i1	2.062572736	9.633717614	2.88E-15	5.55E-14
c141_g1_i1	2.065665759	6.803140214	1.72E-05	0.000109373
c1379_g1_i1	2.08444073	7.888624094	4.58E-09	4.97E-08
c2017_g1_i1	2.0914834	7.354919657	3.28E-07	2.82E-06
c1082_g1_i1	2.123491763	7.265736239	4.92E-07	4.15E-06
c731_g3_i1	2.220046123	6.554090193	3.27E-05	0.000196992
c2364_g1_i1	2.316759772	6.632064529	1.26E-05	8.25E-05
c1690_g1_i1	2.349954887	7.667674037	1.29E-09	1.48E-08
c614_g1_i1	2.36780516	6.153699462	0.000171565	0.000913003
c308_g1_i1	2.426337849	6.919595999	4.98E-07	4.18E-06
c281_g1_i1	2.458484598	6.227652854	7.52E-05	0.000422663
c430_g1_i1	2.503873456	6.78365357	9.54E-07	7.54E-06
c2381_g1_i1	2.592266054	9.555021896	4.56E-21	1.27E-19
c2048_g1_i1	2.695745462	10.17851464	1.85E-25	7.25E-24
c634_g1_i1	2.697347932	9.127580295	6.94E-20	1.77E-18
c387_g1_i1	2.725470257	6.730668951	4.03E-07	3.46E-06
c1080_g1_i1	2.773430637	10.11099734	3.27E-26	1.47E-24
c1409_g1_i1	2.789865983	9.373384291	2.58E-22	8.03E-21
c691_g1_i1	2.926798568	6.896255658	2.19E-08	2.15E-07
c475_g1_i1	2.991546355	7.18392159	5.08E-10	6.07E-09
c412_g1_i1	2.997883873	9.03173465	3.74E-22	1.12E-20
c797_g1_i1	3.011831781	7.992462256	4.35E-15	8.16E-14
c603_g2_i1	3.085484363	6.378693289	1.30E-06	9.97E-06
c8_g1_i1	3.289722524	6.548260144	8.39E-08	7.72E-07
c595_g1_i1	3.323059004	10.44412405	4.74E-36	3.35E-34
c82_g1_i1	3.443769578	6.16740654	1.87E-06	1.38E-05
c568_g2_i1	3.454139899	7.947446284	3.79E-17	8.11E-16
c731_g2_i7	3.461080663	6.181860445	1.90E-06	1.39E-05
c44_g1_i1	3.47274833	6.700897862	6.48E-09	6.76E-08
c2061_g1_i1	3.481056789	9.453202456	2.25E-30	1.16E-28
c59_g1_i1	3.488194709	8.132067384	6.47E-19	1.57E-17
c717_g3_i1	3.502469381	6.725752863	4.39E-09	4.79E-08
c393_g2_i1	3.753156206	5.563439257	8.43E-05	0.00047108
c2423_g1_i1	3.839682917	5.636444203	5.24E-05	0.000304979
c1763_g1_i1	3.849249195	5.644513405	5.24E-05	0.000304979

c1715_g1_i1	3.877341423	13.02295568	7.46E-60	1.05E-57
c404_g1_i1	3.87811195	7.04179233	5.99E-12	8.69E-11
c728_g2_i1	3.932605724	5.714811061	2.21E-05	0.000136144
c731_g2_i4	3.932605724	5.714811061	2.21E-05	0.000136144
c1983_g1_i1	3.932605724	5.714811061	2.21E-05	0.000136144
c1382_g1_i1	3.989946717	5.763157801	1.44E-05	9.32E-05
c289_g1_i1	4.011407991	5.781251245	9.47E-06	6.31E-05
c190_g1_i1	4.137835493	6.750093921	1.30E-10	1.69E-09
c668_g1_i1	4.282499109	6.009814179	7.18E-07	5.85E-06
c302_g1_i1	4.282499109	6.009814179	7.18E-07	5.85E-06
c647_g2_i1	4.445888611	6.147683344	8.84E-08	8.11E-07
c419_g2_i1	4.484767389	6.180513609	6.34E-08	5.94E-07
c2358_g1_i1	4.590408957	8.948913716	1.52E-34	1.01E-32
c673_g1_i1	4.715228179	8.881662748	2.11E-34	1.31E-32
c2357_g1_i1	4.790573222	6.439236503	7.09E-10	8.34E-09
c1730_g1_i1	5.121844275	6.720933974	2.76E-12	4.09E-11
c251_g2_i1	5.132656595	6.730160582	3.19E-12	4.70E-11
c469_g2_i1	5.369150733	7.812270115	1.90E-23	6.49E-22
c1507_g1_i1	5.413754621	9.871877223	6.49E-54	7.64E-52
c422_g1_i1	5.473024541	7.021885478	3.21E-15	6.13E-14
c58_g1_i1	5.529154506	7.0702548	8.77E-16	1.72E-14
c664_g2_i1	5.563085058	7.984095263	4.83E-26	2.13E-24
c1698_g1_i1	5.869658993	9.489644441	1.77E-50	1.98E-48
c409_g2_i1	6.379053291	7.813748439	6.74E-26	2.85E-24
c2345_g1_i1	7.031125861	10.28492445	1.18E-71	2.08E-69
c1087_g1_i1	7.232494315	10.0777035	3.77E-68	6.13E-66
c494_g1_i1	7.732622653	12.52791495	5.60E-123	3.95E-120
c69_g1_i1	8.376491723	11.18757554	3.06E-97	9.25E-95
c224_g1_i1	9.989763963	4.505233102	0.000142624	0.000764756
c667_g2_i1	10.01853319	4.530333755	9.87E-05	0.000542892
c2051_g1_i1	10.02988214	4.540231097	9.87E-05	0.000542892
c349_g3_i1	10.02988214	4.540231097	9.87E-05	0.000542892
c370_g1_i1	10.04114251	4.55004872	9.87E-05	0.000542892
c2390_g1_i1	10.13346509	4.630450045	7.28E-05	0.000410351
c71_g1_i1	10.1646885	4.657604743	5.09E-05	0.000297673
c2071_g1_i1	10.25449808	4.735608693	3.82E-05	0.000228492
c344_g1_i1	10.30207776	4.776873174	1.89E-05	0.000119408
c349_g1_i1	10.37952735	4.84395504	9.37E-06	6.26E-05
c383_g2_i1	10.38395627	4.84778785	1.46E-05	9.38E-05
c367_g1_i1	10.41890655	4.87802187	1.03E-05	6.83E-05
c174_g2_i1	10.4232164	4.88174866	1.03E-05	6.83E-05
c658_g1_i1	10.43606922	4.892860781	7.34E-06	5.03E-05
c596_g1_i1	10.4445746	4.900212674	7.34E-06	5.03E-05
c143_g1_i1	10.5625793	5.002088016	2.95E-06	2.12E-05
c1697_g1_i1	10.71096603	5.129876976	8.87E-07	7.06E-06
c275_g1_i1	10.79651065	5.20339989	5.31E-07	4.44E-06
c1116_g1_i1	10.9626491	5.345917589	6.74E-08	6.28E-07
c2349_g1_i1	11.00063663	5.378458654	7.95E-08	7.36E-07
c344_g3_i1	11.02919197	5.402909762	5.85E-08	5.51E-07
c726_g2_i1	11.03201667	5.405328008	4.31E-08	4.09E-07
c427_g1_i1	11.07920912	5.44571821	5.09E-08	4.81E-07
c563_g2_i1	11.14852587	5.505006229	1.53E-08	1.53E-07
c486_g2_i1	11.26355367	5.603306363	3.16E-09	3.48E-08
c1109_g1_i1	11.3988171	5.718792899	1.14E-09	1.32E-08

c2399_g1_i1	11.56600542	5.861438663	1.50E-10	1.91E-09
c697_g2_i1	11.57571738	5.869723226	1.15E-10	1.51E-09
c1704_g1_i1	11.58344015	5.876310896	8.78E-11	1.16E-09
c468_g2_i1	11.75907819	6.026138664	8.12E-12	1.16E-10
c574_g2_i1	11.88002091	6.129348648	1.86E-12	2.81E-11
c37_g2_i1	11.91103966	6.155829537	6.96E-13	1.08E-11
c868_g1_i1	12.03026014	6.257662711	1.43E-13	2.39E-12
c1106_g1_i1	12.13906668	6.350695862	4.07E-14	7.13E-13
c607_g2_i1	12.34617346	6.52811941	8.46E-16	1.67E-14
c697_g3_i1	12.84812491	6.960861204	3.02E-20	7.91E-19
c607_g1_i1	12.8640643	6.974683271	1.79E-20	4.80E-19
c2416_g1_i1	13.17385552	7.244519548	1.69E-23	5.98E-22
c731_g4_i1	13.78830238	7.787527334	4.28E-31	2.32E-29
c1442_g1_i1	14.01294577	7.988968492	5.08E-34	2.99E-32
c37_g1_i1	14.63713545	8.557403104	9.26E-44	8.17E-42

Supplementary Table S6. The results of *S. aureus* ATCC25923 drug susceptibility tests by broth dilution or disk diffusion methods.

Antibiotics	Zone diameter interpretive criteria nearest whole mm			MIC interpretive critria (μg/mL)			Bacteriostatic circle's diameter in tests (mm)	MIC in the test (μg/mL)	Test results
	S	I	R	S	I	R			
Tetracycline	——[1]	——	——	⩽4	8	⩾16	——	4	sensitive
Gentamicin	——	——	——	⩽4	8	⩾16	——	2	sensitive
Ampicillin	——	——	——	⩽0.25	-	⩾0.5	——	0.25	sensitive
Erythromycin	——	——	——	⩽0.5	1-4	⩾8	——	0.5	sensitive
Vancomycin	——	——	——	⩽2	4-8	⩾16	——	2	sensitive
Oxacillin[2]	——	——	——	⩽2	-	⩾4	——	2	sensitive
Cefoxitin[3] (30μg)	⩾22	-	⩽21	——	——	——	25	——	sensitive

[1] The experiment was not carried.

[2,3] We identified *S. aureus* ATCC25923 as methicillin-susceptible strain based on oxacillin and cefoxitin results.

At least three independent experimets were producted according to methods as recommended by the Clinical and Laboratory Standards Institute (CLSI, M100-S22).

Supplementary Table S7. Primers and conditions used in PCR and qRT-PCR assays.

ID	Gene name	Sequence (5'——3')	Product size (bp)	PCR and qRT-PCR conditions cycling	Reference
Primers for PCR to identify *agr* genotype					
	pan-*agr*	ATGCACATGGTGCACATGC		94°C 5 min; 26 × (94°C 30 s, 55°C 30 s, 72°C 1 min); 72°C 10 min	21
	agr I	GTCACAAGTACTATAAGCTGCGAT	439		
	agr II	GTATTACTAATTGAAAAGTGCCATAGC	573		
	agr III	CTGTTGAAAAAGTCAACTAAAAGCTC	406		
	agr IV	CGATAATGCCGTAATACCCG	657		
Primers for qRT-PCR					
C556_g1_i1	*agr*A-F<br>*agr*A-R	TGATAATCCTTATGAGGTGCTT<br>CACTGTGACTCGTAACGAAAA	164	95°C 5 min; 40 × (95 °C 5 s, annealing 57 °C 30 s, extension 72 °C 30 s)	42,43
C435_g1_i1	*spa*-F<br>*spa*-R	GCAAACGGCACTACTGCTGA<br>CACCAGTTTCTGGTAATGCTTGAG	151		44
C191_g1_i1	*hld*-F<br>*hld*-R	AAGAATTTTTATCTTAATTAAGGAAGGAGTG<br>TTAGTGAATTTGTTCACTGTGTCGA	111		10,45
C501_g1_i1	*ica*R-F<br>*ica*R-R	ATCTAATACGCCTGAGGA<br>TTCTTCCACTGCTCCAA	205	95°C 5 min; 40 × (95 °C 5 s, annealing 53 °C 30 s, extension 72 °C 30 s)	46
C1780_g1_i1	*bbp*-F<br>*bbp*-R	ATTACGGATGATTTCACACCAGTTG<br>TTAAATCAAATGTTCCCTTCGCTATT	76	95°C 5 min; 40 × (95 °C 5 s, annealing 60 °C 30 s, extension 72 °C 30 s)	47
C690_g1_i1	*cna*-F<br>*cna*-R	AATAGAGGCGCCACGACCGT<br>GTGCCTTCCCAAACCTTTTGAGCA	156		48
C1710_g1_i1	*pyk*-F<br>*pyk*-R	GCATCTGTACTCTTACGTCC<br>GGTGACTCCAAGTGAAGA	90	95°C 5 min; 40 × (95 °C 5 s, annealing 50 °C 20 s, extension 72 °C 30 s)	49

References

42. Leng, B. F. *et al.* Allicin reduces the production of alpha-toxin by *Staphylococcus aureus*. *Molecules* **16**, 7958-7968 (2011).

43. Qiu, J. *et al.* Subinhibitory concentrations of perilla oil affect the expression of secreted virulence factor genes in *Staphylococcus aureus*. *PLoS One* **6**, e16160 (2011).

44. Merino, N. *et al.* Protein A-mediated multicellular behavior in *Staphylococcus aureus*. *J Bacteriol* **191**, 832-843 (2009).

45. Delgado, S. *et al.* Characterization of *Staphylococcus aureus* strains involved in human and bovine mastitis. *FEMS Immunol Med Microbiol* **62**, 225-235 (2011).

46. Yu, D., Zhao, L., Xue, T. & Sun, B. *Staphylococcus aureus* autoinducer-2 quorum sensing decreases biofilm formation in an *ica*R-dependent manner. *BMC Microbiol* **12**, 288 (2012).

47. Otsuka, T. *et al.* Key adhesin gene in community-acquired methicillin-resistant *Staphylococcus aureus*. *Biochem Biophys Res Commun* **346**, 1234-1244 (2006).

48. Atshan, S. S. *et al.* Quantitative PCR analysis of genes expressed during biofilm development of methicillin resistant *Staphylococcus aureus* (MRSA). *Infect Genet Evol* **18**, 106-112 (2013).

49. Theis, T., Skurray, R. A. & Brown, M. H. Identification of suitable internal controls to study expression of a *Staphylococcus aureus* multidrug resistance system by quantitative real-time PCR. *J Microbiol Methods* **70**, 355-362 (2007).
